# Estrogen Interactions With Lipid Rafts Related to Neuroprotection. Impact of Brain Ageing and Menopause

**DOI:** 10.3389/fnins.2018.00128

**Published:** 2018-03-06

**Authors:** Raquel Marin, Mario Diaz

**Affiliations:** ^1^Laboratory of Cellular Neurobiology, Department of Basic Medical Sciences, Medicine, Faculty of Health Sciences, University of La Laguna, Tenerife, Spain; ^2^Fisiología y Biofísica de la Membrana Celular en Patologías Neurodegenerativas y Tumorales, Consejo Superior de Investigaciones Cientificas, Unidad Asociada de Investigación, Universidad de La Laguna Tenerife, Tenerife, Spain; ^3^Laboratory of Membrane Physiology and Biophysics, Department of Animal Biology, Edaphology and Geology, University of La Laguna, Tenerife, Spain

**Keywords:** menopause, estrogen receptors, estrogen, lipid rafts, neurodegeneration, signalosome

## Abstract

Estrogens (E2) exert a plethora of neuroprotective actions against aged-associated brain diseases, including Alzheimer's disease (AD). Part of these actions takes place through binding to estrogen receptors (ER) embedded in signalosomes, where numerous signaling proteins are clustered. Signalosomes are preferentially located in lipid rafts which are dynamic membrane microstructures characterized by a peculiar lipid composition enriched in gangliosides, saturated fatty acids, cholesterol, and sphingolipids. Rapid E2 interactions with ER-related signalosomes appear to trigger intracellular signaling ultimately leading to the activation of molecular mechanisms against AD. We have previously observed that the reduction of E2 blood levels occurring during menopause induced disruption of ER-signalosomes at frontal cortical brain areas. These molecular changes may reduce neuronal protection activities, as similar ER signalosome derangements were observed in AD brains. The molecular impairments may be associated with changes in the lipid composition of lipid rafts observed in neurons during menopause and AD. These evidences indicate that the changes in lipid raft structure during aging may be at the basis of alterations in the activity of ER and other neuroprotective proteins integrated in these membrane microstructures. Moreover, E2 is a homeostatic modulator of lipid rafts. Recent work has pointed to this relevant aspect of E2 activity to preserve brain integrity, through mechanisms affecting lipid uptake and local biosynthesis in the brain. Some evidences have demonstrated that estrogens and the docosahexaenoic acid (DHA) exert synergistic effects to stabilize brain lipid matrix. DHA is essential to enhance molecular fluidity at the plasma membrane, promoting functional macromolecular interactions in signaling platforms. In support of this, DHA detriment in neuronal lipid rafts has been associated with the most common age-associated neuropathologies, namely AD and Parkinson disease. Altogether, these findings indicate that E2 may participate in brain preservation through a dual membrane-related mechanism. On the one hand, E2 interacting with ER related signalosomes may protect against neurotoxic insults. On the other hand, E2 may exert lipostatic actions to preserve lipid balance in neuronal membrane microdomains. The different aspects of the emerging multifunctional role of estrogens in membrane-related signalosomes will be discussed in this review.

## General overview

Sex steroids produced by peripheral glands such as dihydrotestosterone (DHT), testosterone (T), estradiol (E2), progesterone (PROG), and corticosterone (CORT) are traditionally known to play crucial roles in sexually dimorphic circuits located in the hypothalamus and other brain areas controlling reproductive behaviors, as well as brain masculinization, brain connectivity, and neuroplasticity (Losecaat Vermeer et al., 2016; Panzica and Melcangi, 2016). Furthermore, the central and peripheral nervous systems show local synthesis of neurosteroids (Melcangi et al., 2008; Giatti et al., 2010). These neuroactive steroids are considered important regulators of neural functions, including brain and cerebellar development, hippocampal neuritogenesis and synaptogenesis (Murakami et al., 2017), and neuroprotection against numerous pathologies, such as Alzheimer's disease (AD), Parkinson disease (PD), Huntington's disease (HD), stroke, multiple sclerosis (MS), spinal cord injury, peripheral neuropathy, and psychiatric disorders (Melcangi et al., 2016). These disorders show gender differences in their incidence and progression, as an indicative of the different roles of neurosteroids in the physiological actions in the nervous system. Indeed, sex hormones also influence the local concentrations of neurosteroids that affect the pathological context. Thus, epidemiological studies have demonstrated a higher incidence in women of AD, HD, MS, peripheral neuropathy, and some psychiatric disorders, such as anxiety, depression, and eating disorders. In the contrary, men show a higher incidence of PD, stroke and autism as compared to women (Andersen et al., 1999; Wooten et al., 2004; Afifi, 2007; Reeves et al., 2008; Melcangi et al., 2016). In particular related to AD, neuroprotective effects of estrogens in either cellular, animal, and clinical studies have been extensively studied and characterized, whereas brain beneficial actions of androgens and stress steroids have been less investigated, and require further clarification of the common molecular mechanisms of neurosteroid effects. Interestingly, the incidence of AD in men does not seem directly related to estrogens, since estrogens in men do not exhibit a reduction with aging. Rather, detriment in testosterone levels in men brain may predict enhanced vulnerability to AD (Moffat et al., 2004; Pike, 2017). This phenomenon appears to be sex-specific, since testosterone levels do not show significant changes in women suffering AD (Rosario et al., 2011).

A general clinical observation is that menopausal women show a higher risk of developing a neurodegenerative disease, indicating that estrogens are neuroprotective. Numerous data in the last decade have concluded that estrogens (E2) exert a plethora of neuroprotective actions against aged-associated brain diseases, including AD. Part of these actions takes place through binding to estrogen receptors (ER) embedded in signalosomes, where numerous signaling proteins are clustered. Signalosomes are preferentially located in lipid rafts which are dynamic membrane microstructures characterized by a peculiar lipid composition enriched in gangliosides, saturated fatty acids, cholesterol, and sphingolipids. Rapid E2 interactions with ER-related signalosomes appear to trigger intracellular signaling ultimately leading to the activation of molecular mechanisms against AD.

We have previously observed that the reduction of E2 blood levels occurring during menopause induced disruption of ER-signalosomes at frontal cortical brain areas. These molecular changes may reduce neuronal protection activities, as similar ER signalosome derangements were observed in AD brains. The molecular impairments may be associated with changes in the lipid composition of lipid rafts observed in neurons during menopause and AD. These observations indicate that the changes in lipid raft structure during aging may be at the basis of alterations in the activity of ER and other neuroprotective proteins integrated in these membrane microstructures.

Moreover, E2 is a homeostatic modulator of lipid rafts. Recent work has pointed to this relevant aspect of E2 activity to preserve brain integrity, through mechanisms affecting lipid uptake and local biosynthesis in the brain. Some findings have demonstrated that estrogens and the docosahexaenoic acid (DHA) exert synergistic effects to stabilize brain lipid matrix. DHA is essential to enhance molecular fluidity at the plasma membrane, promoting functional macromolecular interactions in signaling platforms. In support of this, DHA detriment in neuronal lipid rafts has been associated with the most common age-associated neuropathologies, namely AD and Parkinson disease.

Altogether, these findings indicate that E2 may participate in brain preservation through a dual membrane-related mechanism. On the one hand, E2 interacting with ER related signalosomes may protect against neurotoxic insults. On the other hand, E2 may exert lipostatic actions to preserve lipid balance in neuronal membrane microdomains. The different aspects of the emerging multifunctional role of estrogens in membrane-related signalosomes will be discussed in this review.

### Neurological transition during menopause

Perimenopause and menopause are important periods of woman's life where significant physiological changes occur. Perimenopause is a period in women whose age range is between 40–58 years, followed by a transition period of 1–5 years that culminates with ~12 months of amenorrhoea (Harlow et al., 2012). Menopause is the final stage associated with cessation of ovarian secretion of female sex hormones, estrogen, and progesterone (Greendale et al., 2013). It is calculated that, within EEUU and Europe, ~ >176 million women undergo menopausal periods, and this number increases by 9 million per year. Moreover, worldwide, it is estimated that there are >850 million women in perimenopause stages (Brinton et al., 2009). Even more, only 20% of women are asymptomatic during this process, whereas the remaining 80% suffer different symptoms associated with hormone detriment.

Even though perimenopausal stages are focused on changes in the reproductive system by oocyte depletion (hot flushes, vaginal dryness, fatigue, irregular periods, etc.), the majority of symptoms of these periods are neurological in nature (Brinton et al., 2015). These features are variable, and appear to be co-incident with a decline of brain metabolism (Rasgon et al., 2005). The most common neurological disturbances reported are insomnia, mood changes, depression, subjective memory complaints, and cognitive dysfunction (specifically learning and memory impairments).

Several studies have documented that women show higher protection against the nervous system pathologies as compared with men, whereas this tendency is inverted after menopause (Sherwin and Henry, 2008; Pike, 2017). Consequently, it is plausible that falling estrogen levels during menopausal periods may increase the risk of neuronal vulnerability against injury. In agreement with this, ovariectomy before natural menopause is correlated with a higher incidence of dementia and PD (Rocca et al., 2008). Also, premature menopausal women show a higher risk of AD (Ryan et al., 2014). Furthermore, numerous evidence has agreed that the progression of menopause increases the incidence of neurological perturbations associated with the most common aged-related neurodegenerative diseases, such as AD, and PD, as well as cerebral stroke, ischemia and multiple sclerosis (Ramagopalan et al., 2010; Schreihofer and Ma, 2013; Picillo et al., 2017; Pike, 2017).

Besides gonadal hormone detriment in menopause, synthesis of estrogens at, both, central and peripheral nervous systems may also be affected with the progression of aging (Melcangi et al., 2008; Giatti et al., 2015). Thus, neurosteroidogenesis is decreased during menopause (Rosario et al., 2011), and this reduction is exacerbated in AD brains (Schumacher et al., 2003). Furthermore, the link between estrogen loss in post-menopausal women and the risk of dementia is supported by clinical evidence reporting that the incidence of AD is 2–3 times higher in women than in men (Ryan et al., 2014). Overall, multifactorial variations of estrogenic production in, both, gonadal and nervous system during menopausal periods may intervene in the resulting neurological impairments. Moreover, these data reflect the importance of developing novel accurate hormonal replacement strategies to counteract the potential cognitive decline related to menopause, despite the inconclusive and discouraging results obtained in previous clinical trials (Merlo et al., 2017).

### Estrogens protect the brain

Estrogens display a variety of physiological roles in the brain, including neuronal differentiation, neurogenesis, and neuronal plasticity, which are crucial for brain homeostasis, cognition, and preservation (Brinton, 2009; Engler-Chiurazzi et al., 2017). In addition, E2 regulates actions on glial cells at, both, central and peripheral nervous system (CNS and PNS) including oligodendroglia, astrocytes, and microglia (CNS), and Schwann cells (PNS). These hormone actions include remyelination, inflammation, edema formation, and extracellular glutamate levels which are important in the regulation of physiological homeostasis, and preservation against pathophysiological situations (Arevalo et al., 2010). Different data has reported that Schwann cells, oligodendrocytes and astrocytes are targets of E2. Thus, the hormone enhances myelin sheet formation and the synthesis of myelin basic protein through direct actions in Schwann and oligodendroglial cells (Jung-Testas et al., 1992, 1993; Fex Svenningsen and Kanje, 1999; Marin-Husstege et al., 2004). E2 also regulates the morphology of astrocytes as well as the expression of numerous molecules involved in the regulation of astrocytic functions (Luquin et al., 1993; Garcia-Segura et al., 1996; McCarthy, 2008). Furthermore, E2 modulates microglial response to inflammation, thus avoiding overreaction of these cells following brain injury (Vegeto et al., 2006; Tapia-Gonzalez et al., 2008).

Taking into account these data, it is plausible to affirm that menopause-related alterations in the nervous system may be highly associated with estrogen depletion and estrogen receptors (ERs) regulation. A big body of data has reported a number of mechanisms by which estrogens (in particular, 17β-estradiol, E2) protect against different neuroinflammatory and neurodegenerative disorders. Thus, E2 has been demonstrated to exert beneficial actions against a wide range of diseases: AD, PD, ischemia, schizophrenia, multiple sclerosis, hypertensive encephalopathy, spinal cord injury, traumatic brain injury, and retinal degeneration (De Nicola et al., 2012; Petrone et al., 2014; Cascio et al., 2015; Lan et al., 2015; Brotfain et al., 2016; Itoh et al., 2017; McGregor et al., 2017; Raghava et al., 2017). Furthermore, a plethora of *in vivo* and *in vitro* studies over more than two decades have provided evidence that estrogen exerts beneficial effects against different insults (Brann et al., 2007; Petrovska and Jurisic, 2012). Among other toxic paradigms, it has been documented that estrogens protect neurons against glutamate excitotoxicity, glucose and serum deprivation, stress injury, hydrogen peroxide, iron, sodium azide, and Aβ- and MPTP-induced toxicities (Siddiqui et al., 2016). In addition, E2 contributes to modulate the decrease in gliotic responses under neurodegenerative conditions, through different actions including glial cell proliferation after brain injury (Garcia-Estrada et al., 1993; Zhang et al., 2002; Vegeto et al., 2006). In particular in AD, E2 enhances Aβ uptake by microglia, as a mechanism to promote Aβ clearance (Li et al., 2000; Yue et al., 2005).

The precise molecular mechanisms underlying E2 neuroprotective effect still remain elusive because of the vast complexity of the brain. Even though some of these actions may be explained by the intrinsic antioxidant properties of this hormone, acting as free radical scavenger of oxidative stress (Prokai et al., 2003), the majority of E2 neuroprotective effects require binding to ERs. To date, three distinct ERs have been characterized distinctly distributed throughout the different brain areas: ERα, ERβ, and G-protein coupled ER1 (GPER) (Prossintz and Barton, 2011; Lu and Herndon, 2017). In addition, a variety of splice variants of ERα and ERβ (ranging from 36 to 80 kDa) has also been identified in different systems, although its functional relevance in brain preservation is still unclear (Ascenzi et al., 2006; Marin et al., 2006; Kim et al., 2017). Interestingly, certain of these splice forms are brain-area specific. In this sense, an ERα-splice variant (MB1) has been shown to increase its expression in women brains during the transition period to menopause, as an indicator of their potential role during aging (Ishunina and Swaab, 2008).

Furthermore, ERα and ERβ have also been detected in glial cells. Thus, some immunohistochemical assays have demonstrated an abundant localization of ERs in glial cells of rat and mouse brain (Cardona-Gómez et al., 2000; Quesada et al., 2007; Sierra et al., 2008; Tapia-Gonzalez et al., 2008). Both ERs show partially distinct distribution in neural tissues, suggesting that they may have distinct or complementary actions that modulate glial responses related to remyelination, anti-inflammatory process, edema formation, and other reparative mechanisms (Arevalo et al., 2010). Thus, ERβ participates in preservation of axonal integrity and demyelination in oligodendrocytes (Tiwari-Woodruff and Voskuhl, 2009), and against ischemia in microglia in the hippocampus (Takahashi et al., 2004). Expression of this receptor in oligodendroglia has also been recently shown to play a role in optic glioma-induced retinal dysfunction (Toonen et al., 2017). Furthermore, ERα, but not ERβ, expressed in oligodendrocytes plays anti-inflammatory actions in an animal model of multiple sclerosis (Tiwari-Woodruff et al., 2007). ERα has also been detected in microglia from mouse adult brain and rat cerebellum following peripheral inflammation (Sierra et al., 2008; Tapia-Gonzalez et al., 2008). Moreover, both receptors play a role throughout neural tissue maturation showing a differential expression in astrocytes and oligodendrocytes in the spinal cord during rat development (Platania et al., 2003). Collectively, these results suggest that glial cells utilize E2 bound to ERs to influence reparative mechanisms within microenvironments in the brain and SNP.

## Multifactorial roles of estrogen receptors in brain preservation

ERs are widely distributed in different brain areas. These receptors are highly expressed in the hypothalamus, which is a primary center regulator of body temperature, sleep, and circadian rhythms (McEwen et al., 2012). ERs are also shown in important regions for memory, cognition, attention, sensory integration, mood, emotion, and motivation, such as the prefrontal cortex, hippocampus, amygdala, posterior cingulate, thalamus, raphe nucleus, and locus coeruleus (McEwen et al., 2012; Brinton et al., 2015; Hara et al., 2015). The distribution patterns of ERα and ERβ are distinct, observing higher levels of ERα in hypothalamus and amygdala, whereas ERβ is most abundant in the thalamus and hippocampus (Osterlund et al., 2000a,b), as an indicative of different roles developed by these receptors in the brain.

The neurobiological circuits expressing ERs are affected during the perimenopausal transition, in co-incidence with the most common neurological symptoms of perimenopausal periods. Brain changes in ER patterns have been investigated in aged female primates as a valuable model for studying the menopause-related alterations that may affect brain functionality (Gilardi et al., 1997; Walker and Herndon, 2008). During menopause in rhesus monkey females, a lower density of synapse spines, and changes in synapsis structure have been observed, in particular, in the hippocampal dentate gyrus and prefrontal cortex (Hara et al., 2015). These changes were correlated with lower memory performance, whereas the cognitive skills were recovered by estrogen treatment in the menopausal monkeys (Hara et al., 2014). Reduction in hippocampal synaptic density was also observed in a mouse model of menopause (Van Kempen et al., 2014), suggesting a role of E2 in neuronal plasticity.

A high number of evidence has demonstrated that E2 bound to its receptor follows different pathways that are subjected to multifactorial extracellular and intracellular events. With the exception of GPER that binds to the hormone at the plasma membrane, ERα and ERβ are dynamic molecules that have been shown to be located within distinct neuronal and glial compartments suggesting the co-existence of different intracellular mechanisms of E2 action (Milner et al., 2001; Pawlak et al., 2005; Ogiue-Ikeda et al., 2008). In the cellular nucleus, E2 binding to ERs induces in a few hours genomic (or classical) mechanisms of action, leading to the transcriptional activation of late response genes that regulate, among others, apoptosis, and inflammation (Marin et al., 2005; Heldring et al., 2007). Other ERs, ERβ in particular, are found in mitochondria, contributing to maintain mitochondrial functionality (Nilsen et al., 2007; Yang et al., 2009). In these organelles, E2 acts as regulator of bioenergetics circuits (Brinton, 2008), counteracting the oxidative stress and glucose hypometabolism etiologies implicated in AD and PD, as well as amyotrophic lateral sclerosis (ALS) (Simpkins and Dykens, 2008).

Furthermore, a wide variety of neuroprotective actions has been shown to occur within seconds to minutes following E2 exposure. These actions are referred to as rapid or non-genomic mechanisms, where plasma membrane-associated ERs binding to the hormone are involved (Pietras and Szego, 1977; Levin, 2009). These non-genomic mechanisms promote the rapid activation of different intracellular signaling pathways that ultimately may lead to neuroprotection. The best characterized pathway in neurons involves the activation of extracellular signal-regulated kinases (ERK) and phosphotidylinositol 3-kinase (PI3K)/Akt/glycogen synthase kinase 3 (GSK3) pathway in brain areas related to memory and cognition (Kelly and Levin, 2001; Marin et al., 2005; Garcia-Segura et al., 2006; Sheppard et al., 2017). In septal and hippocampal neurons, activation of PI3K/Akt/GSK3 signal transduction has been shown to protect against injuries induced by, Aβ exposure, glutamate exocytosis, staurosporine-induced apoptosis, and oxygen glucose deprivation (Marin et al., 2005; Zhao et al., 2016). Apart from neuronal survival, this signaling pathway mediates cytoskeletal remodeling, synaptic plasticity, and traumatic brain injury (Garcia-Segura et al., 2007; Wang et al., 2017). Another preferential alternative intracellular pathway by E2 membrane interactions involve activation of Raf/MEK/ERK signaling, which enhances neuroprotection following ischemic brain injury, stroke and Aβ- and glutamate-induced toxicities (Bryant et al., 2006; Lebesgue et al., 2009).

Although still not fully characterized, the generally held view maintains that these membrane-related mechanisms of E2 are modulated by cell membrane homeostasic mechanisms, where plasma membrane functional microdomains may play a preferential role.

## Involvement of neuronal lipid rafts in rapid estrogen signaling

Lipid rafts are dynamic membrane microstructures enriched in distinct lipid classes, such as cholesterol, sphingolipids, saturated fatty acids, and gangliosides (Lingwood and Simons, 2010). This peculiar molecular composition confers particular physico-chemical properties, observing a higher molecular order and microviscosity as compared to non-raft membrane regions. Lipid rafts are regulators of signaling platforms (or signalosomes) formed by subsets of proteins that compartmentalize in multimolecular clusters to trigger different cellular responses (Levental and Veatch, 2016). The association of apparent hydrophobic proteins to lipid rafts typically takes place through association with different structural features or lipid moieties that confer stability to the molecular complexes. The most common protein anchoring targets in these microstructures are glycosylphosphatidylinositol (GPI), cholesterol, glycosphingolipids (GPL) *S*-palmitoylation, *N*-myristoylation/palmitoylation, and *S*-acylation with saturated fatty acids (Fantini, 2007; Levental et al., 2010). Moreover, the particular microstructure of these highly molecular-ordered domains may have consequences in the configuration of proteins embedded. The molecular rearrangements may favor the interaction of protein entities that co-exist in the same raft microdomain, thereby enhancing the formation of functional multiprotein clusters.

The subgroup of membrane-related ERs (mERs) classically represents a small fraction of the total amount of receptors within the cell. ER molecule lacks either transmembrane domains, hydrophobic residues or other structural modifications to be inserted into the plasma membrane (Pedram et al., 2007). Although the strategies developed by a subpopulation of ERs to anchor into the lipid bilayer are not yet fully clarified, some results in different cell types have demonstrated that palmitoylation of the receptor is required to be trafficked to the cell membrane (Meitzen et al., 2013). This modification lies in the covalent attachment of palmitic acid and other fatty acids to a cysteine residue present in ERs to increase hydrophobicity. In particular, Cys447 located in the ligand binding domain has been demonstrated to be essential for ERα to interact with the cell membrane. Indeed, the replacement of this aminoacid by Ala447 abrogates receptor insertion into the membrane compartment (Acconcia et al., 2005).

The other necessary requirement for membrane trafficking of ERs is their association with lipid rafts (Marin et al., 2012; Maselli et al., 2015). In these microdomains, ERα also appears to be palmitoylated (Liu et al., 2002). The stability of this receptor in lipid rafts is achieved by its interaction with caveolin-1, a raft scaffolding protein that allows ERα membrane anchoring (Boulware et al., 2007). In support of this, ERα primary structure shows a consensus sequence at positions 463–470 of the ligand binding domain susceptible of binding to caveolar scaffolding domain (CSD) present in caveolin-1 (Marin et al., 2008). This consensus motif is conserved in different proteins involved in signal transduction (Couet et al., 1997), and is required for the transport of signaling proteins into raft domains (Massimino et al., 2002). Furthermore, ERα downstream signaling may also involve other members of the caveolin family, such as caveolin-2 and -3, as demonstrated in neurons from different brain regions (Micevych and Mermelstein, 2008).

Lipid rafts provide the proper microenvironment for the recruitment and integration of a wide range of receptors within signaling platforms (signalosomes) that are activated upon specific extracellular stimulation, thereby inducing distinct cell responses. In this scenario, numerous lipid raft-associated proteins have been shown to be involved in nervous system functioning. The list includes GPI-anchored receptors; G protein-coupled receptors (adrenergic receptors, adenosine receptors and cannabinoid receptors); glutamate receptors (AMPA, NMDA, mGluR); neurotrophin receptors (tyrosine kinase receptors, TrkA, TrkB, ephrin receptor, Eph, c-Ret, ErbB); Src family receptors (c-Src, Lyn, Fyn); cell adhesion molecules (NCAMs, TAG-1, Thy-1); and proteins associated with myelin glycosynapse (LINGO1, p75, NgR1, myelin-associated glycoprotein). The association of this plethora of proteins in signalosomes has been shown to modulate synapsis, neuronal plasticity, cell-cell communication, myelin organization and stability, autophagy, neuronal survival, and neurodegeneration (George and Wu, 2012; Egawa et al., 2016). For an excellent review of the importance of the lipid raft-related protein classes in the brain context see Sonnino et al. (2014).

Several lines of evidence have demonstrated that ERs, ERα in particular, form part of raft-integrated signalosomes to initiate a variety of neuronal responses by mechanisms still not fully elucidated (Srivastava et al., 2011). Caveolin-1 has been shown to be the pivotal docking protein of ERα-related signalosomes in brain areas related to memory and cognition. Thus, caveolin scaffolding protein serves to determine interactions of ER with metabotropic glutamate receptors (mGluRs) in lipid rafts of the hippocampus and striatum (Meitzen and Mermelstein, 2011; Micevych and Kelly, 2012). E2 signaling initiated at the membrane level by interaction with the pairing of mGluR and ER triggers intracellular responses that may be important for, both, neuronal and glial physiology. This membrane estrogen signaling occurs in the absence of mGluR-specific ligands, and represents one of the underlying mechanisms of rapid estrogen actions related to the nervous system functioning. Moreover, caveolin-1 is also the pivotal anchor of ERα interactions with the insulin growth factor-1 receptor β (IGF-1Rβ). This ERα/IGF-1Rβ tandem has been shown to be cross-talked modulated by their natural ligands, E2 and IGF-1, which mutually cooperate in the prevention of age-related neuronal dysfunction (Alonso and Gonzalez, 2012; Arevalo et al., 2015), and are crucial in brain preservation against AD (Marin, 2011; Lan et al., 2015).

It has been postulated that part of E2 neuroprotective actions triggered in ERα signalosomes against Aβ toxicity occur by the activation of different signal transduction pathways, including a voltage-dependent anion channel (VDAC) gating modulation. This channel appears associated with ERα in neuronal lipid rafts from a wide variety of brain regions, including septum, hippocampus, and cortex from, both, murine and human origins (Marin et al., 2007, 2009; Ramirez et al., 2009), where it participates in different pathogenesis including AD (Thinnes, 2015). In raft fractions of cortical neurons, VDAC interacting with Aβ promotes the channel dephosphorylation in tyrosine residues, a phenomenon that promotes VDAC gating, and enhances neuronal death (Fernandez-Echevarria et al., 2014). Indeed, VDAC appears in a dephosphorylating status in cortical raft fractions of AD brains at late stages, as an indicative of the toxic post-transductional modification of the channel in correlation with the pathology (Canerina-Amaro et al., 2017). Conversely, E2 binding to ERα signalosome has been shown to prevent VDAC channel dephosphorylation in neurons, as a mechanism underlying cell survival against Aβ neurodegeneration (Herrera et al., 2011a; Thinnes, 2013). This hormonal mechanism takes place through activation of Src-kinase and protein kinase A (PKA) signaling pathways (Herrera et al., 2011b). However, other kinases such as c-Jun N-terminal kinase-3 (JNK3) have been shown to regulate mitochondrial VDAC phosphorylation in the brain, thereby affecting the channel conductance and opening probability (Gupta, 2017). Overall, these data indicate that E2 modulation of VDAC phosphorylation in neuronal lipid rafts may be physiologically relevant in brain preservation. In support of this, a significant VDAC dephosphorylation has been observed in lipid rafts from cortical brain areas of menopausal women, in correlation with E2 detriment occurring during this period of women's life. Thus, in lipid rafts isolated from frontal cortex of women above 65 years old, post-transcriptional VDAC pattern was resolved in two main isoforms corresponding to non-phosphorylated forms as compared to samples from women younger than 55 years old (Canerina-Amaro et al., 2017). Similar results were obtained in AD samples from women at late stages of the disease, detecting a displacement to non-raft fractions of VDAC in parallel with dephosphorylation of the porine. The trafficking of VDAC out of lipid raft microdomains was accompanied by an impairment of ER-related signaling complex. These phenomena may have important consequences for cell preservation, as E2 phosphorylation of VDAC in lipid rafts is an important prerequisite to palliate Aβ-induced neurotoxicity (Herrera et al., 2011a; Fernandez-Echevarria et al., 2014). Indeed, selective estrogen receptor modulators (SERMs) such as tamoxifen show the opposite effects than the hormone, thus enhancing VDAC dephosphorylation (Herrera et al., 2011b) and gating (Valverde et al., 2002). Overall, although still inconclusive, these data indicate that hormone alterations in cortical post-menopausal lipid rafts may contribute to a progressive deterioration of neuronal functionality and survival through deregulation of VDAC.

Moreover, emerging evidence indicates a potential role of E2 in the lipid homeostatic preservation of the neuronal membrane, which is crucial to maintain stability of functional signalosomes.

## Lipid raft alterations in neurodegenerative diseases

The brain is highly enriched in functional lipids and, consequently, the lipid homeostasis is particularly important in this organ. Given that lipid raft structure and activity require a particular proportion of distinct lipid classes, it follows that alterations in the lipid content in these microdomains can lead to abnormal functioning that may contribute to neuropathological events. Some results have reported that loss of lipid raft integrity correlates in general with brain aging progression. Different events may induce lipid impairment in these membrane structures, such as detriment in the lipid amount (either by intake or local bioynthesis), alterations in the proportion of polyunsaturated fatty acids (PUFA), increase in saturate/unsaturated ratio, and decrease in ganglioside or cholesterol levels that induce cell aging and death (Ledesma et al., 2012; Colin et al., 2016). It has been postulated that raft changes in lipid profile may induce modifications in the biophysical properties of these microstructures (e.g., peroxidability, viscosity, thermodynamics) that may contribute to neuronal detriment in cognitive brain areas (Diaz et al., 2015). Thus, in mice brains, subtle changes in the molecular composition of lipid rafts undergo an “aging” process throughout normal life that produces, among other events, an increase in membrane-order and reduction in the peroxidability index, notably impacting the lateral organization of these microstructures (Diaz et al., 2012). Indeed, these aberrant features are aggravated by AD-like genotype, observing an acceleration of lipid raft aging in parallel with AD progression (Fabelo et al., 2012b). Furthermore, alterations of lipid raft lipid matrix have been observed in age-associated neuropathologies even at pre-symptomatic stages, such as AD, PD, and dementia of Lewy bodies (DLB), suggesting that lipid raft impairment may be an early parameter of neuropathology (Fabelo et al., 2011, 2014; Marin et al., 2017). These changes are summarized in Table 1. Related to these dementias, the most significant lipid variations are the reduced levels of cholesterol, gangliosides, PUFA, plasmalogens, cerebrosides, and sulfatides as compared with age-matched controls (Molander-Melin et al., 2005; Han, 2007; Fabelo et al., 2011; Ariga, 2017; Marin et al., 2017). Anomalies in lipid metabolism of lipid rafts have also been reported in other neurological diseases, such as Huntington's disease, where a marked reduction of ganglioside levels is observed in striatum and caudate regions which has been related to neuronal apoptosis (Desplats et al., 2007; Valencia et al., 2010). These observations suggest that targeted lipid variations in lipid raft normal molecular composition may be early events to progressive neuronal degeneration. Consequently, identification of these molecular alterations in cell membranes may predict future pathological outcome (Marin et al., 2013a).

**Table 1:** Lipid alterations in neuronal lipid raft microdomains.

**Lipid alteration**	**Neuronal tissue**	**Related anomalies and injury**	**References**
- Reduced levels of PUFA (DHA, AA), and oleic acid - Increased levels of the proportion of saturated fatty acids vs. PUFA	Frontal cortex	Alzheimer's disease at late stages (ADV-VI)	Martín et al., 2010
- Reduced levels of PUFA (DHA, AA), oleic acid, and cerebrosides - Decreased levels of cholesterol and sphingomyelin	Frontal cortex	Low levels of estrogen in menopause Alzheimer's disease at late stages (ADV-VI)	Canerina-Amaro et al., 2017
- Lower levels of cholesterol, sterol esters, sulfatides, and PUFA (DHA, AA) - Increased levels of sphingomyelin and saturated fatty acid content, and increased phospholipids/cholesterol ratio - The changes were highly significant in aged (14 months old) mice	Neocortex	Double-transgenic APP/presenilin mice	Fabelo et al., 2012a
- Higher concentrations of gangliosides GM1 and GM2 - Lower concentrations of cholesterol	Temporal cortex	Early and late stages of Alzheimer's disease	Molander-Melin et al., 2005
- Alterations in the levels of gangliosides - Increased levels of GM1, GM2, GM3, GM4, GD3 - Reduced levels of GD1b and GT1b	Frontal cortex Temporal cortex Parietal cortex Hippocampus Basal telencephalon	Alzheimer's disease and its mice models	Reviewed in Ariga (2017)
- Lower levels of ganglioside GM1 - Higher levels of ganglioside GM3	Cortical areas	Parkinson disease	Di Pasquale et al., 2010
- Reduced levels of cholesterol, gangliosides, PUFA (DHA, AA), plasmalogens, cerebrosides and sulfatides - Higher levels of saturated fatty acids (16:0 and 18:0)	Frontal cortex	Incidental Parkinson disease Parkinson disease	Fabelo et al., 2011
- Low levels of PUFA (DHA), plasmalogens and cholesterol	Frontal cortex	Dementia with Lewy bodies	Marin et al., 2017
- Alterations in ganglioside profiles - Decreased levels of ganglioside GM1 - Increased levels of GD3	Human caudate region Forebrain of R6/1 transgenic mice	Huntington's disease	Desplats et al., 2007
- Increased levels of glucosylceramide, hexosylsphingosine, bis(monoacylglycero)phosphate and gangliosides - Decreased levels of cholesterol and phosphatidylcholine - Altered sphingolipid/cholesterol proportion	Occipital cortex from sheep	Neuronopathic Gaucher disease	Hein et al., 2017

A consequence of lipid instability of membrane microdomains is the alteration in the functionality, molecular interactions and trafficking of proteins integrated in signalosomes. Firstly, the increased in viscosity and membrane order of these microstructures may reduce the lateral mobility and phase transition, thereby affecting lipid and protein interactions (Molander-Melin et al., 2005; Diaz et al., 2015). Secondly, proteins can be misfolded and adopt abnormal configurations that may lead to toxic aggregates and dysfunctional intracellular signaling. Thus, mounting evidence suggests that the key self-aggregating proteins in different proteinopathies, such as Aβ in AD, alpha-synuclein (α-syn) in PD, and prion protein (PrPc) in prion diseases share similar biophysical properties that may affect their biochemical interrelations with membrane-integrated molecular compounds (Goedert, 2015; Ugalde et al., 2016). Noticeably, lipid rafts are considered key sites in the modulation of amyloid-like seeding (Kazlauskaite et al., 2003; Arbor et al., 2016), α-syn pathological fibrillation (Ariga, 2014), and the conversion of PrPc to the scrapie form PrPsc (Taylor and Hooper, 2006). Thirdly, lipid rafts may act as membrane molecular sorting sites that coordinate the spatiotemporal rearrangement of signalosomes according to extracellular ligands availability (Simons and Gerl, 2010). As an example, changes in membrane cholesterol levels, that affect raft microstructure, can result in the stimulation of apoptotic events through activation or deactivation of different raft protein markers, such as receptors and channels (George and Wu, 2012). Taking into account the high number of signaling proteins intrinsically present in lipid rafts, it is conceivable that the consequences of abnormal lipid homeostasis may affect crucial functions such as synapsis, neuroplasticity, and cell preservation (Paratcha and Ibáñez, 2002; Tsui-Pierchala et al., 2002; Sebastião et al., 2013; Egawa et al., 2016). Fourthly, changes in lipid raft microenvironment may alter protein translocation to lipid rafts, thereby modifying their properties. According to this premise, some data support the concept that changes in lipid homeostasis and protein trafficking may underpin the etiology of AD. Notably, the regulation of electrogenic molecules involves their translocation to lipid rafts (Pristerá and Okuse, 2012). Also, accumulation and interaction of the key proteins involved in Aβ processing, the amyloid precursor protein (APP) and β-secretase (BACE) is promoted by lipid alterations in raft microdomains (Parsons and Austen, 2007; Vetrivel et al., 2009). Additionally, this mechanistic pathological processing is initiated in cortical brain areas since the first AD stages (ADI-II) before senile plaques are evidenced, as a prelude to the typical anatomopathological events of this dementia (Fabelo et al., 2014). Moreover, although still not fully clarified, changes in raft lipid content observed in incidental PD and other synucleopathies may promote α-syn toxic structural conformations (Samuel et al., 2016; Marin et al., 2017).

In correlation with this evidence, it is plausible that ER actions integrated in lipid raft platforms may be affected by variations in membrane lipid microenvironment that may ultimately alter estrogen signaling and cell responses (Marin, 2011). In agreement with this, lipid raft molecular analysis in cortical areas of post-menopausal women have shown some changes in the levels of cholesterol, cerebrosides, sterol esters, and PUFA as compared to younger women controls (Canerina-Amaro et al., 2017). Interestingly, a similar trend was reported in cortical lipid rafts from women with AD, detecting an exacerbation of normal lipid composition in these microdomains (Martín et al., 2010). Indeed, the biochemical structure of lipid rafts in cortical areas appears to be sufficient to discriminate between pre- and post-menopausal women, observing in the latter group closer similarities to lipid profiles than those characterized in lipid rafts from AD brains. Furthermore, these changes are known to affect the physico-chemical properties of lipid rafts that may subsequently alter the proteins integrated in these domains. In this order of ideas, ERα-related signalosome was altered in post-menopausal brains, observing a displacement out of the raft of, both, ERα and IGF-1Rβ as a consequence of caveolin-1 dissociation (Marin et al., 2008; Canerina-Amaro et al., 2017). Taking into consideration the requirement of caveolin-1 to initiate E2 signal transduction at the membrane level (Boulware et al., 2007; Luoma et al., 2008), these data suggest that ER-signalosome disruption may affect neuroprotective intracellular responses. In this sense, ERα/IGF-1Rβ/caveolin-1 disarrangement also enhanced the redistribution of VDAC to non-raft fractions, in parallel with a dephosphorylation of the channel, which may increase neuronal vulnerability (Canerina-Amaro et al., 2017). Interestingly, ERα-signalosome disarrangements are exacerbated in cortical and hippocampal lipid rafts of AD brains (Ramirez et al., 2009), thereby supporting the relationship between membrane ERα-complex modifications and the process of aging and cognitive decline.

Overall, anomalies in lipid raft matrix appear to be an early event in neurodegenerative processes by modifying membrane protein clustering that regulates intracellular physiological responses. Consequently, preservation of membrane lipid homeostasis may be a key factor for preventing or decelerating neuronal dysfunction. In this sense, an emerging role of E2 has been associated with brain lipid balance (Pellegrini et al., 2014). These aspects are discussed in the next section.

## Estrogen as lipostatic agent in neuronal membranes

Emerging data suggest that E2 may play a role in lipid homeostatic balance of lipid rafts (Marin et al., 2013b; Maselli et al., 2015). These actions may take place through cross-talk interactions between the hormone and distinct lipid classes that play an important role in these microstructures' dynamics, such as PUFA and cholesterol. In this sense, based in nutrigenomic approaches, cholesterol and PUFA diets affect the expression of several genes involved in lipid raft formation (Puskas and Kitajka, 2006). Although still little explored, these estrogenic activities at the cell membrane may be highly relevant regarding E2-related activities for brain preservation.

The docosahexaenoic acid (DHA) is a PUFA highly abundant in the human brain (25–30% of total fatty acids), where it is a major component of cell membrane phospholipids. Paradoxically, the brain has very poor capacity to produce DHA (Pawlosky et al., 2001; Barceló-Coblijn and Murphy, 2009), which is a main limiting factor to ensure an adequate supply of this fatty acid to the nervous system. DHA plays a crucial role in proper brain development and function (Calder, 2017). It has been reported that a deficit of this PUFA increases the risk of cognitive impairment and dementia, in particular, AD and PD (Söderberg et al., 1991; Bazan et al., 2011; Salem et al., 2015; Colin et al., 2016; Sun et al., 2017). Brain beneficial actions of DHA take place at different levels, including: (i) Variations in membrane fluidity, permeability and elasticity due to its unsaturated conformation (Rawicz et al., 2000; Stillwell and Wassall, 2003); (ii) Involvement in intracellular signaling and apoptosis, and generation of neuroprotectins (Bazan, 2003, 2006); (iii) Modulation of membrane protein functioning (Calder, 2016); (iv) Regulation of the antioxidant system factors (Casañas-Sánchez et al., 2014, 2015). In particular, DHA appears to confer resilience against AD development rather than a general effect throughout the brain, a phenomenon that may be related to the higher DHA turnover in regions involved in synaptogenesis and synapsis plasticity in learning and memory activities (Denis et al., 2013; Yassine et al., 2016). Moreover, although not particularly abundant in lipid rafts, DHA has a significant impact in the structure of these microdomains, due in part to the effects of this PUFA on cholesterol distribution (Wassall and Stillwell, 2009). In this order of ideas, reduction of DHA content in parallel with structural modifications of lipid rafts have been reported in different dementias, such as AD, PD, and DLB (Fabelo et al., 2011, 2014; Marin et al., 2017). DHA detriment occurring with aging may be partially explained by dietary deficits that may progressively affect the incorporation of this essential fatty acid into the membrane phospholipids, which are mainly replaced by monounsaturated fatty acids (Fabelo et al., 2012b, 2014). Thus, DHA supplementation has been associated with lower cerebral amyloidosis, higher cognitive, and memory performance, emotional disturbances, and cerebral vasculature improving during brain aging (Yurko-Mauro et al., 2010; Vellas et al., 2014; Boespflug et al., 2016).

An additional important factor in DHA turnover is associated with E2 regulation at the brain level. It is worth mentioning that, in addition to gonadal production, there is a local production of estrogens in the hippocampus (Galea et al., 2006; Hojo et al., 2008; Barker and Galea, 2009) although, at present, no clear demonstrations have shown a reduction of endogenous neurosteroids related to aging (Overk et al., 2013). Therefore, it is plausible that estrogen effects in DHA homeostasis may be the result of, both, local and peripheral estrogen origins. In this sense, it has been demonstrated that DHA plasma levels are about 15% higher in women than in men following similar control diets. Administration of oral ethinyl estradiol in women increased DHA by 42%. On the contrary, testosterone administrated to female-to-male transsexual subjects provoked a significant decrease (by 22%) in DHA concentrations (Giltay et al., 2004). These data indicate that E2, but not testosterone may enhance the synthesis of DHA from the diet precursor α-linolenic acid (ALA). This biosynthesis takes place predominantly in the liver, which counts on the expression of the different elongases and desaturases needed to produce this PUFA (Cho et al., 1999). Thus, these data indicate that the greater capacity of women to convert ALA to DHA than do men (Burdge and Wootton, 2003) is related to E2 hormone. This E2 enhancer effect in DHA production may have important consequences on homeostasis of this fatty acid in the brain. Notably, despite the high abundance of DHA in the brain, this organ has a very low capacity to endogenous synthesis of this fatty acid (Plourde and Cunnane, 2007; Brenna et al., 2009). Yet it is generally accepted that neuron and glial cells possess the genetic machinery to synthesize *de novo* saturated and monosaturated fatty acids, and nerve cells are also endowed with the enzymes to produce PUFA (Bazinet and Layé, 2014). However, PUFA content in the brain is mainly provided by the blood, and the rate of PUFA uptake into the nervous system is much higher than the local production. Thus, *in vivo* studies in humans have demonstrated that only a 0.5% of ALA is converted to DHA (Plourde and Cunnane, 2007). Moreover, even though PUFA dietary supply appears to upregulate the expression of the enzymes involved in DHA synthesis in the liver, in contrast, enzyme levels remain static in the brain (Rapoport, 2013). In agreement with this, it has been recently reported that dietary DHA supplementation in mice does not significantly increase the local expression of the elongases and desaturates involved in DHA synthesis in the hippocampus (Díaz et al., 2016). Collectively, these results indicate that DHA content in the brain depends on a constant supply from the peripheral blood.

Based upon these observations, it is plausible that the combinatory effects of E2 and DHA factors may have significant consequences in nerve cell biology and brain preservation. Likely, the best characterized evidence of the DHA and E2 interplay in humans is the demonstration of a higher prevalence rate of depression following DHA deficiency and ovarian hormonal dysregulation (Davis et al., 2010). Indeed, it has been reported a synergistic antidepressant action of DHA and E2 in the regulation of serotonergic neurotransmission through brain-derived neurotrophic factor (BDNF) and inflammatory cytokines (Jin and Park, 2015). These antidepressant-like effects were specific of DHA since its precursor alpha-linolenic acid did not show any effect in serotonergic circuits (Choi and Park, 2017).

Moreover, the combination of DHA-enriched diets and E2 treatment appears to be a key factor in maintaining lipid homeostasis in the hippocampus (Díaz et al., 2016). These E2-related lipostatic mechanisms involve the genetic regulation of lipid biosynthetic pathways, which are crucial for hippocampal maintenance against AD phenotype in mice, in particular, at early stages of this pathology (Marin et al., 2013b; Díaz et al., 2016). Other evidence of the importance of E2 in DHA bioavailability in the brain has been reported during pregnancy, where there is a preferential high demand of DHA for the fetal brain formation. In this instance, it has been demonstrated that E2 is the main factor of brain DHA uptake in both the maternal and fetal brain, with a higher production of PUFA in the maternal liver also observed (Fabelo et al., 2012a). These data suggest that the hormone may play a role in, both, brain intake and brain production of DHA. Other experiments in cultured cells support the idea that E2 upregulates the metabolic production of DHA from its precursors. Thus, *in vitro* experiments in human neuroblastoma SHSY-5Y cells have reported that these cells maintain the ability to convert a certain proportion of ALA to DHA. Interestingly, E2, but not dehidroepiandrosterone, upregulates the DHA production (by 50%), and its uptake into the plasma membrane (Alessandri et al., 2008). This E2 mechanism involves the modulation of the Delta5-desaturase expression (Extier et al., 2009). These observations suggest that E2 may be an enhancer of the neuronal endogenous production of this PUFA, and may satisfy to a certain degree meet the neuronal membrane demands of DHA. However, the highest proportion of DHA present in neuronal membranes comes from diet sources. Furthermore, E2 and DHA modulation in the brain may be dual, since dietary DHA intake conversely potentiates E2 synthesis in the cerebral cortex, a factor that is required to delay the onset and elongate the latency of epileptic seizures (Ishihara et al., 2017). Overall, these studies reveal the synergistic efficacy of E2 and DHA in physiological actions in the brain.

A big body of data has reported the role of membrane cholesterol in the neuroprotective effects of E2 (Peri et al., 2011). The brain contains about 25% of the total amount of unesterified cholesterol content in the total body. Therefore, it is plausible that cholesterol embedded into the neuronal membranes, and not just its peripheral levels, may be a key factor for brain maintenance and functionality (Yanagisawa, 2002). In this scenario, it has been reported that cholesterol plays a multifactorial role in cell membranes. Thus, this molecule is a key factor for lipid rafts microstructure and functionality (Egawa et al., 2016), and its optimal amount may create a protected barrier against toxic factors (Arispe and Doh, 2002). Some studies also reported that raft cholesterol may have a role in the biogenesis and catabolism of β-amyloid (Araki and Tamaoka, 2015). In particular in lipid rafts, β-amyloid formation may be influenced by cholesterol turnover that affects the fluidity and structural properties of these microdomains. Thus, a loss of neuronal membrane cholesterol facilitates β-amyloid formation machinery (Abad-Rodriguez et al., 2004). In accordance with this, low cholesterol levels have been detected in cortical lipid rafts from AD patients in parallel with increased levels of steryl/cholesteryl esters (Martín et al., 2010).

Estrogens may play a preferential role for the control of cholesterol synthesis and trafficking in the neuronal membranes. Recent findings have indicated that the production of this fatty acid is stimulated by estrogens. Some data has reported that E2 affects cholesterol synthesis in the hippocampus, through the modulation in expression of 3-hydroxy-3-methylglutaryl coenzyme A reductase (HMG-CoAR) and acetyl-coenzyme A acetyltransferase (ACAT) genes (Marin et al., 2013b; Díaz et al., 2016). This action may be achieved by the intervention of seladin-1 (for SELective Alzheimer's Disease INdicator-1), a molecule that plays a dual role as a neuroprotector agent as well as a catalyzer of cholesterol formation from its precursor desmosterol (Peri, 2016).

Another important level of interaction of E2 and lipid transport in the brain takes place through the expression of apolipoprotein ApoE. This lipoprotein is a crucial regulator of cholesterol metabolism in the brain. Different ApoE forms (ApoE2, ApoE3, and ApoE4) have been characterized. In this sense, ApoE4 is the greatest genetic risk factor to develop sporadic AD, and this risk is greater in women than men (Neu et al., 2017). In particular, (Apo)E3 isoform is stimulated by this hormone, to facilitate neurite outgrowth (Nathan et al., 2004). Distinct regulation of ApoE isoforms by estrogens may have an important role in neuroprotection against AD, since hormonal administration at menopause have benefits in ApoE2 and ApoE3 production in women by decreasing extracellular and soluble β-amyloid (Kunzler et al., 2014). These observations suggest a distinct E2 modulation of Apo subtypes, which may have important consequences in neuroprotection against AD (Shi et al., 2014).

Overall, these data reflect the importance of E2 in lipid synthesis and uptake in the brain throughout women's lifespan that can be affected as a consequence of hormonal changes.

## Future perspectives

**Figure 1:**
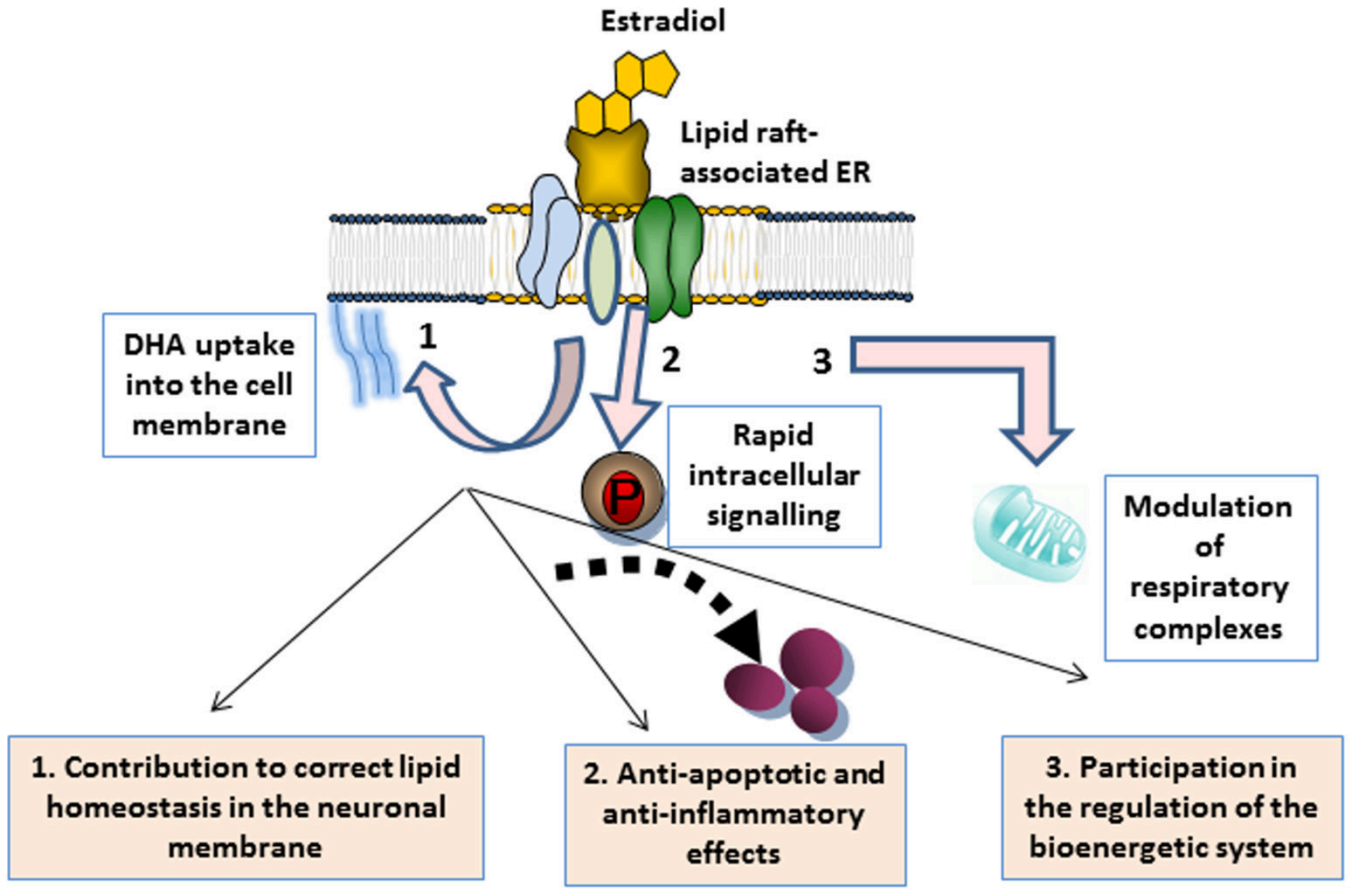
Schematic representation of multiple actions of estrogen associated with neuronal membrane microdomains. **1** Estrogen binding to ERs integrated in lipid raft signalosomes triggers the rapid activation of rapid signal transduction, ultimately leading to the modulation of either anti-apoptotic or anti-inflammatory factors that contribute to neuronal maintenance. **2** The hormone also contributes to membrane lipid homeostasis, such as DHA membrane uptake, thus promoting healthy protein clustering and activities. **3** Estrogen is also an energetic and metabolic capacitor, through the participation in the mitochondrial membrane turnover and the regulation of mitochondrial supercomplexes that regulate the cell bionergetic system.

As discussed throughout the different sections, a growing body of evidence supports that E2 has a beneficial impact in the brain by exerting multiple actions that work in conjunction with neuronal membrane microenvironments. These actions are summarized in Figure 1. In plasma membrane lipid rafts, E2 plays a dual action. On the one hand, it targets ERα clustered in multimeric signalosomes formed by different molecules that trigger neuroprotective signal transduction. On the other hand, it contributes to the maintenance of the proper lipid environment to promote healthy molecular interactions for neuronal functioning. In particular, evidence indicates that E2 modulates cell membrane intake of cholesterol and DHA abundantly represented in the nervous system, and significantly contribute to lipid raft structure (Su, 2010; Peri et al., 2011; Marin et al., 2013b). Preservation of lipid homeostasis in these microdomains appear to be crucial for protein stability and interactions in signaling platforms, but it may also contribute to enhance neuronal defenses against oxidative stress (Casañas-Sánchez et al., 2014). It is worth mentioning that, although still not fully demonstrated, steroid hormone contribution to membrane molecular turnover may also take place at the mitochondrial membrane level. Thus, lipid rafts have also been identified in mitochondria, where they have important implications in optimal assemblies of respiratory supercomplexes and apoptosis regulation (Garofalo et al., 2015). In these organelles, lipid rafts may participate in energetic and metabolic capacity (Ray et al., 2016). Moreover, some reports have indicated that particular lipid features of lipid rafts may promote specific protein assemblies for mitochondrial functioning. In this order of ideas, it has been demonstrated that DHA content modulates the molecular architecture of cardiolipin-protein scaffolds, which are the pivotal structure of mitochondrial lipid rafts (Shaikh et al., 2015). Therefore, it is conceivable that lipid anomalies in lipid rafts induced by estrogenic fluctuations may converge in reduced energy production and cell exhaustion (Ferrer, 2009). However, this possibility remains to be confirmed.

Given that E2 detriment occurring during menopause enhances the risk of neuropathological events, estrogen replacement therapy (ERT) may be considered a logical intervention. Nevertheless, ERT approaches are still a matter of controversy, because of the increased rates of stroke, coronary heart disease, and breast cancer reported in menopausal women following this preventive therapy (Prentice, 2014). A major reason for these unsuccessful trials is lack of standard criteria and optimization of the parameters for accurate hormonal treatments. For instance, there remains much to be learned concerning the optimal treatment guidelines regarding formulation, dose and timing of intervention to avoid unfavorable health consequences (Manson et al., 2013). However, taking into account the potential benefits of this type of therapies in nervous system preservation, further research to determine the best ERT strategy is crucial, although remains still pending.

Some promising data accrued from human studies have provided evidence that estrogen replacement therapies (ERT) administrated to women at 50–63 years old might protect against cognitive decline occurring during normal aging (Henderson, 2014). Thus, it has been demonstrated that ERT improves performance on tests of verbal, visual working and spatial memory, and verbal fluency, as compared to non-users women of similar age (Robinson et al., 1994; Kimura, 1995; Grodstein et al., 2000; Miller et al., 2002; Sherwin and Henry, 2008). However, there are multiple factors such as the particular physiological features of each subject (age, health, hormone status, etc.) that make ERT unsuitable for some women in long-term basis (Marjoribanks et al., 2017). In addition, there is not still consensus about the efficacy of hormone treatments in the brain due to multifactorial parameters, including E2 origin, dose, and timing of duration that may largely influence either efficiency or adverse risk factors (Hogervorst et al., 2000). In this sense, a proposed alternative strategy is the development of efficacious NeuroSERM (specific SERMs designed for the brain) that may avoid the peripheral adverse effects in other E2-targeted organs (Zhao et al., 2005). However, this endeavor requires a better knowledge of the orchestrated estrogen mechanisms of action at the different subcellular compartments in nerve cells.

An alternative intervention that appears to alleviate the impact of metabolic changes during menopausal periods is based on nutritional supplementation with vegetable phytoestrogens (Villa et al., 2017). Phytoestrogens have similar structures to those of estrogen, possess estrogen-like activities, and show some affinity for ERs (Brzezinski and Debi, 1999). Consequently, supplementation with the most common phytoestrogens, such as isoflavones (i.e., genistein), and stilbenes (i.e., resveratrol) is a remedy used for a significant number of women to alleviate some menopausal symptoms (Soni et al., 2014). Isoflavones are abundant in soybean products (e.g., tofu), and resveratrol is highly abundant in grape skin and berries. Interestingly, some evidence indicates that supplementation with either isoflavones or resveratrol to menopausal women in early stages (<10 years) show some beneficial effects in memory and cognition, as well as improved cerebral vascularization (Evans et al., 2016; Thaung Zaw et al., 2017).

Furthermore, emerging data discussed in this review have demonstrated a synergistic effect of, both, E2 and essential fatty acids such as DHA in the maintenance of neural homeostasis and preservation against neurodegeneration (Fabelo et al., 2012a; Díaz et al., 2016). Based on these premises, future potential interventions may include supplementation of, both, hormonal treatments and selected lipid classes that may have beneficial effects during menopausal periods. In particular, specific nutritional supplements enriched in omega-3 fatty acids abundantly found in fish oil (i.e., DHA) combined with phytoestrogens may provide protection against cognitive aging. In this sense, some promising studies in cancer cellular models have indicated that diets rich in fish oils and soy isoflavones may be a good complementary treatment against breast cancer (Duncan et al., 2005), and osteoporosis (Kruger et al., 2015). Indeed, the combination of these two compounds appear to reduce breast cancer risk by enhancing anti-inflammatory pathways and lowering the pro-inflammatory effects induced by prostanoids, cyclooxygenase-2 (COX-2), and arachidonic acid (AA) activities (Horia and Watkins, 2007). In agreement with this, other data have also shown that reduced levels of DHA as a consequence of diets poor in this PUFA promote inflammation in distinct neuropathologies (Sinclair et al., 2007; Zárate et al., 2017). Moreover, the fact that studies in experimental mouse models of AD have demonstrated a deleterious effect in the hippocampus of low levels of, both, E2 and DHA, also supports the existence of a PUFA/hormone synergistic neuroprotective effect (McNamara et al., 2009). However, to the best of our knowledge, no studies have addressed the potential beneficial effects of dual supplementary intake of PUFA and phytoestrogen in human nervous system.

In conclusion, we believe that nutritional-hormonal interventions may be a potential therapeutic strategy to alleviate some of the symptoms associated with, both, menopause and age-related brain degeneration, particularly at the stage of earliest recognizable symptoms. These strategies may act as compensatory mechanisms to palliate the loss of essential cell membrane lipids that ultimately may lead to neuronal dysfunction and brain detriment. Notwithstanding these encouraging results, further studies will be needed to tackle optimal strategies to mitigate menopausal effects in the nervous system.

## Author contributions

RM is the main contributor to this work; RM and MD have written the main body of the text, and provided the content of the different sections of this review.

### Conflict of interest statement

The authors declare that the research was conducted in the absence of any commercial or financial relationships that could be construed as a potential conflict of interest.

## References

[B1] Abad-Rodriguez J. Ledesma M. D. Craessaerts K. Perga S. Medina M. Delacourte A.. (2004). Neuronal membrane cholesterol loss enhances amyloid peptide generation. J. Cell Biol. 167, 953–960. 10.1083/jcb.200404149 15583033 PMC2172459

[B2] Acconcia F. Ascenzi P. Bocedi A. Spisni E. Tomasi V. Trentalance A.. (2005). Palmitoylation-dependent estrogen receptor αmembrane localization: regulation by 17β estradiol. Mol. Biol. Cell. 16, 231–237. 10.1091/mbc.E04-07-0547 15496458 PMC539167

[B3] Afifi M. (2007). Gender differences in mental health. Singapore Med. J. 48, 385–391. Available online at: http://smj.sma.org.sg/4805/4805ra1.pdf 17453094

[B4] Alessandri J. M. Extier A. Langelier B. Perruchot M. H. Heberden C. Guesnet P.. (2008). Estradiol favors the formation of eicosapentaenoic acid (20:5n-3) and n-3 docosapentaenoic acid (22:5n-3) from alpha-linolenic acid (18:3n-3) in SH-SY5Y neuroblastoma cells. Lipids 43, 19–28. 10.1007/s11745-007-3117-6 17912567

[B5] Alonso A. Gonzalez C. (2012). Neuroprotective role of estrogens: relationship with insulin/IGF-1 signaling. Front. Biosci. 4, 607–619. 10.2741/e403 22201898

[B6] Andersen K. Launer L. J. Dewey M. E. Letenneur L. Ott A. Copeland J. R.. (1999). Gender differences in the incidence of AD and vascular dementia: the EURODEM Studies. EURODEM Incidence Research Group. Neurology 53, 1992–1997. 10.1212/WNL.53.9.1992 10599770

[B7] Araki W. Tamaoka A. (2015). Amyloid β-protein and lipid rafts: focused on biogenesis and catabolism. Front. Biosci. 20, 314–324. 10.2741/4311 25553453

[B8] Arbor S. C. LaFontaine M. Cumbay M. (2016). Amyloid-beta Alzheimer targets – protein processing, lipid rafts, and amyloid-beta pores. Yale J. Biol. Med. 89, 5–21. 27505013 PMC4797837

[B9] Arevalo M. A. Azcoitia I. Gonzalez-Burgos I. Garcia-Segura L. M. (2015). Signaling mechanisms mediating the regulation of synaptic plasticity and memory by estradiol. Horm. Behav. 74, 19–27. 10.1016/j.yhbeh.2015.04.016 25921586

[B10] Arevalo M. A. Santos-Galindo M. Bellini M. J. Azcoitia I. Garcia-Segura L. M. (2010). Actions of estrogens on glial cells: implications for neuroprotection. Biochim. Biophys. Acta 1800, 1106–1112. 10.1016/j.bbagen.2009.10.002 19818384

[B11] Ariga T. (2014). Pathogenic role of ganglioside metabolism in neurodegenerative diseases. J. Neurosci. Res. 92, 1227–1242. 10.1002/jnr.23411 24903509

[B12] Ariga T. (2017). The pathogenic role of ganglioside metabolism in Alzheimer's Disease-cholinergic neuron-specific gangliosides and neurogenesis. Mol. Neurobiol. 54, 623–638. 10.1007/s12035-015-9641-0 26748510

[B13] Arispe N. Doh M. (2002). Plasma membrane cholesterol controls the cytotoxicity of Alzheimer's disease AβP., (1-40) and., (1-42) peptides. FASEB J. 16, 1526–1536. 10.1096/fj.02-0829com 12374775

[B14] Ascenzi P. Bocedi A. Marino M. (2006). Structure-function relationship of estrogen receptor α and β: impact on human health. Mol. Aspects Med. 27, 299–402. 10.1016/j.mam.2006.07.001 16914190

[B15] Barceló-Coblijn G. Murphy E. J. (2009). α-linolic acid and its conversion to longer chain n-3 fatty acids: benefits for human health and a role in maintaining tissue n-3 fatty acid levels. Prog. Lipid Res. 48, 355–374. 10.1016/j.plipres.2009.07.002 19619583

[B16] Barker J. M. Galea L. A. (2009). Sex and regional differences in estradiol content in the prefrontal cortex, amygdala and hippocampus of adult male and female rats. Gen. Comp. Endocrinol. 164, 77–84. 10.1016/j.ygcen.2009.05.008 19457436

[B17] Bazan N. G. (2003). Synaptic lipid signaling: significance of polyunsaturated fatty acids and platelet-activating factor. J. Lipid Res. 44, 2221–2233. 10.1194/jlr.R300013-JLR200 13130128

[B18] Bazan N. G. (2006). Cell survival matters: docosahexaenoic acid signaling, neuroprotection and photoreceptors. Trends Neurosci. 29, 263–271. 10.1016/j.tins.2006.03.005 16580739

[B19] Bazan N. G. Molina M. F. Gordon W. C. (2011). Docosahexaenoic acid signalolipidomics in nutrition: significance in aging, neuroinflammation, macular degeneration, Alzheimer's, and other neurodegenerative diseases. Annu. Rev. Nutr. 31, 321–321. 10.1146/annurev.nutr.012809.104635 21756134 PMC3406932

[B20] Bazinet R. P. Layé S. (2014). Polyunsaturated fatty acids and their metabolites in brain function and disease. Nat. Rev. Neurosci. 15, 771–785. 10.1038/nrn3820 25387473

[B21] Boespflug E. L. McNamara R. K. Eliassen J. C. Schidler M. D. Krikorian R. (2016). Fish oil supplementation increases event-related posterior cingulate activation in older adults with subjective memory impairment. J. Nutr. Health Aging 20, 161–169. 10.1007/s12603-015-0609-6 26812512 PMC12877669

[B22] Boulware M. I. Kordasiewicz H. Mermelstein P. G. (2007). Caveolin proteins are essential for distinct effects of membrane estrogen receptors in neurons. J. Neurosci. 27, 9941–9950. 10.1523/JNEUROSCI.1647-07.2007 17855608 PMC6672640

[B23] Brann D. W. Dhandapani K. Waked C. Mahesh V. B. Khan M. M. (2007). Neurotrophic and neuroprotective actions of oestrogen: basic mechanisms and clinical implications. Steroids 72, 381–405. 10.1016/j.steroids.2007.02.003 17379265 PMC2048656

[B24] Brenna J. T. Salem N. Jr. Sinclair A. J. Cunnane S. C. International Society for the Study of Fatty Acids, and Lipids, ISSFAL. (2009). α-Linolenic acid supplementation and conversion to n-3 long-chain polyunsaturated fatty acids in humans. Prostaglandins Leukot. Essent. Fatty Acids. 80, 85–91. 10.1016/j.plefa.2009.01.004 19269799

[B25] Brinton R. D. (2008). The healthy cell bias of estrogen action: mitochondrial bioenergetics and neurological implications. Trends Neurosci. 10, 529–537. 10.1016/j.tins.2008.07.003 PMC10124615 18774188

[B26] Brinton R. D. (2009). Estrogen-induced plasticity from cells to circuits: predictions for cognitive function. Trends Pharmacol. Sci. 30, 212–222. 10.1016/j.tips.2008.12.006 19299024 PMC3167490

[B27] Brinton R. D. Gore A. C. Schmidt P. J. Morrison J. H. (2009). Mammalian hormone-behavior systems, in Hormones, Brain and Behaviour, 2nd Edn., eds Pfaff D. W. Arnold A. P. Fahrbach S. E. Etgen A. M. Rubin R. T. (Philadelphia, PA: Elsevier), 2199–2222.

[B28] Brinton R. D. Yao J. Yin F. Mack W. J. Cadenas E. (2015). Perimenopause as a neurological transition state. Nat. Rev. Endocrinol. 11, 393–404. 10.1038/nrendo.2015.82 26007613 PMC9934205

[B29] Brotfain E. Gruenbaum S. E. Boyko M. Kutz R. Zlotnik A. Klein M. (2016). Neuroprotection by estrogen and progesterone in traumatic brain injury and spinal cord injury. Curr. Neuropharmacol. 14, 641–653. 10.2174/1570159X14666160309123554 26955967 PMC4981744

[B30] Bryant D. N. Sheldahl L. C. Marriott L. K. Shapiro R. A. Dorsa D. M. (2006). Multiple pathways transmit neuroprotective effects of gonadal steroids. Endocrine 29, 199–207. 10.1385/ENDO:29:2:199 16785596

[B31] Brzezinski A. Debi A. (1999). Phytoestrogens: the “natural” selective estrogen receptor modulators? Eur. J. Obstet. Gynecol. Reprod. Biol. 85, 47–51. 10.1016/S0301-2115(98)00281-4 10428321

[B32] Burdge G. C. Wootton S. A. (2003). Conversion of α-linolenic acid to palmitic, palmitoleic, stearic and oleic acids in men and women. Prostaglandins Leukot. Essent. Fatty Acids 69, 283–290. 10.1016/S0952-3278(03)00111-X 12907139

[B33] Calder P. C. (2016). Docosahexaenoic acid. Ann. Nutr. Metab. 69, 7–21. 10.1159/000448262 27842299

[B34] Calder P. C. (2017). Very long-chain n-3 fatty acids and human health: fact, fiction and the future. Proc. Nutr. Soc. 17, 1–21. 10.1017/S0029665117003950 29039280

[B35] Canerina-Amaro A. Hernandez-Abad L. G. Ferrer I. Quinto-Alemany D. Mesa-Herrera F. Ferri C.. (2017). Lipid raft ER signalosome malfunctions in menopause and Alzheimer's disease. Front. Biosci. 9, 111–126. 10.2741/s476 27814578

[B36] Cardona-Gómez G. P. DonCarlos L. Garcia-Segura L. M. (2000). Insulin-like growth factor I receptors and estrogen receptors colocalize in female rat brain. Neuroscience 99, 751–760. 10.1016/S0306-4522(00)00228-1 10974438

[B37] Casañas-Sánchez V. Pérez J. A. Fabelo N. Herrera-Herrera A. V. Fernández C. Marín R. (2014). Addition of docosahexaenoic acid, but not arachidonic acid, activates glutathione and thioredoxin antioxidant systems in murine hippocampal HT22 cells: potential implications in neuroprotection. J. Neurochem. 131, 470–483. 10.1111/jnc.12833 25060706

[B38] Casañas-Sánchez V. Pérez J. A. Fabelo N. Quinto-Alemany D. Díaz M. L. (2015). Docosahexaenoic., (DHA) modulates phospholipid-hydroperoxide glutathione peroxidase., (Gpx4) gene expression to ensure self-protection from oxidative damage in hippocampal cells. Front. Physiol. 6:203. 10.3389/fphys.2015.00203 26257655 PMC4510835

[B39] Cascio C. Deidda I. Russo D. Guarneri P. (2015). The estrogenic retina: the potential contribution to healthy aging and age-related neurodegenerative diseases of the retina. Steroids 103, 31–41. 10.1016/j.steroids.2015.08.002 26265586

[B40] Cho H. P. Nakamura M. Clarke S. D. (1999). Cloning, expression, and fatty acid regulation of the human delta-5 desaturase. J. Biol. Chem. 274, 37335–37339. 10.1074/jbc.274.52.37335 10601301

[B41] Choi J. E. Park Y. (2017). EPA and DHA, but not ALA, have antidepressant effects with 17β-estradiol injection via regulation of a neurobiological system in ovariectomized rats. J. Nutr. Biochem. 49, 101–109. 10.1016/j.jnutbio.2017.07.012 28915388

[B42] Colin J. Gregory-Pauron L. Lanhers M. C. Claudepierre T. Corbier C. Yen F. T.. (2016). Membrane raft domains and remodeling in aging brain. Biochimie 130, 178–187. 10.1016/j.biochi.2016.08.014 27594339

[B43] Couet J. Li S. Okamoto T. Ikezu T. Lisanti M. P. (1997). Identification of peptide and protein ligands for the caveolin-scaffolding domain. Implications for the interaction of caveolin with caveolae-associated proteins. J. Biol. Chem. 272, 6525–6533. 10.1074/jbc.272.10.6525 9045678

[B44] Davis P. F. Ozias M. K. Carlson S. E. Reed G. A. Winter M. K. McCarson K. E.. (2010). Dopamine receptor alterations in female rats with diet-induced decreased brain docosahexaenoic acid., (DHA): interactions with reproductive status. Nutr. Neurosci. 13, 161–169. 10.1179/147683010X12611460764282 20670471 PMC2955509

[B45] De Nicola A. F. Brocca M. E. Pietranera L. Garcia-Segura L. M. (2012). Neuroprotection and sex steroid hormones: evidence of estradiol-mediated protection in hypertensive encephalopathy. Mini Rev. Med. Chem. 12, 1081–1089. 10.2174/138955712802762121 22827218

[B46] Denis I. Potier B. Vancassel S. Heberden C. Lavialle M. (2013). Omega-3 fatty acids and brain resistance to ageing and stress: body of evidence and possible mechanisms. Ageing Res. Rev. 12, 579–594. 10.1016/j.arr.2013.01.007 23395782

[B47] Desplats P. A. Denny C. A. Kass K. E. Gilmartin T. Head S. R. Sutcliffe J. G.. (2007). Glycolipid and ganglioside metabolism imbalances in Huntington's disease. Neurobiol. Dis. 27, 265–277. 10.1016/j.nbd.2007.05.003 17600724 PMC2082128

[B48] Di Pasquale E. Fantini J. Chahinian H. Maresca M. Taïeb N. Yahi N. (2010). Altered ion channel formation by the Parkinson's-disease-linked E46K mutant of α-synuclein is corrected by GM3 but not by GM1 gangliosides. J. Mol. Biol. 397, 202–218. 10.1016/j.jmb.2010.01.046 20114052

[B49] Diaz M. L. Fabelo N. Marin R. (2012). Genotype-induced changes in biophysical properties of frontal cortex lipid raft from APP/PS1 transgenic mice. Front. Physiol. 3:454. 10.3389/fphys.2012.00454 23205014 PMC3506919

[B50] Díaz M. Fabelo N. Casañas-Sánchez V. Marin R. Gómez T. Quinto-Alemany D. (2016). Hippocampal lipid homeostasis in APP/PS1 mice is modulated by a complex interplay between dietary, DHA, and Estrogens: relevance for Alzheimer Disease. J Alzheimers Dis. 49, 459–481. 10.3233/JAD-150470 26519437

[B51] Diaz M. Fabelo N. Martín V. Ferrer I. Gómez T. Marin R. (2015). Biophysical alterations in lipid rafts from human cerebral cortex associate with increased BACE1/AβPP interaction in early stages of Alzheimer's Disease. J. Alzheimers. Dis. 43, 1185–1198. 10.3233/JAD-141146 25147112

[B52] Duncan R. E. El-Sohemy A. Archer M. C. (2005). Regulation of HMG-CoA reductase in MCF-7 cells by genistein, EPA, and DHA, alone and in combination with mevastatin. Cancer Lett. 224, 221–228. 10.1016/j.canlet.2004.11.031 15914273

[B53] Egawa J. Pearn M. L. Lemkuil B. P. Patel P. M. Head B. P. (2016). Membrane lipid rafts and neurobiology: age-related changes in membrane lipids and loss of neuronal function. J. Physiol. 594, 4565–4579. 10.1113/JP270590 26332795 PMC4983616

[B54] Engler-Chiurazzi E. B. Brown C. M. Povroznik J. M. Simpkins J. W. (2017). Estrogens as neuroprotectants: estrogenic actions in the context of cognitive aging and brain injury. Prog. Neurobiol. 157, 188–211. 10.1016/j.pneurobio.2015.12.008 26891883 PMC4985492

[B55] Evans H. M. Howe P. R. Wong R. H. (2016). Clinical evaluation of effects of chronic resveratrol supplementation on cerebrovascular function, cognition, mood, physical function and general well-being in postmenopausal women-rationale and study design. Nutrients 8:150. 10.3390/nu8030150 27005658 PMC4808879

[B56] Extier A. Perruchot M. H. Baudry C. Guesnet P. Lavialle M. Alessandri J. M. (2009). Differential effects of steroids on the synthesis of polyunsaturated fatty acids by human neuroblastoma cells. Neurochem. Int. 55, 295–301. 10.1016/j.neuint.2009.03.009 19576517

[B57] Fabelo N. Martin V. González C. Alonso A. Diaz M. (2012a). Effects of oestradiol on brain lipid class and fatty acid composition: comparison between pregnant and ovariectomised oestradiol-treated rats. J. Neuroendocrinol. 24, 292–309. 10.1111/j.1365-2826.2011.02242.x 22007691

[B58] Fabelo N. Martín V. Marín R. Moreno D. Ferrer I. Díaz M. (2014). Altered lipid composition in cortical lipid rafts occurs at early stages of sporadic Alzheimer's disease and facilitates APP/BACE1 interactions. Neurobiol. Aging 35, 1801–1812. 10.1016/j.neurobiolaging.2014.02.005 24613671

[B59] Fabelo N. Martín V. Marín R. Santpere G. Aso E. Ferrer I.. (2012b). Evidence for premature lipid raft aging in APP/PS1 double-transgenic mice, a model of familial Alzheimer disease. J. Neuropathol. Exp. Neurol. 71, 868–881. 10.1097/NEN.0b013e31826be03c 22975585

[B60] Fabelo N. Martín V. Santpere G. Marín R. Torrent L. Ferrer I.. (2011). Severe alterations in lipid composition of frontal cortex lipid rafts from Parkinson's disease and incidental Parkinson's disease. Mol. Med. 17, 1107–1118. 10.2119/molmed.2011.00119 21717034 PMC3188884

[B61] Fantini J. (2007). Interaction of proteins with lipid rafts through glycolipid-binding domains: biochemical background and potential therapeutic applications. Curr. Med. Chem. 14, 2911–2917. 10.2174/092986707782360033 18045136

[B62] Fernandez-Echevarria C. Díaz M. Ferrer I. Canerina-Amaro A. Marin R. (2014). Aβ promotes VDAC1 channel dephosphorylation in neuronal lipid rafts. Relevance to the mechanisms of neurotoxicity in Alzheimer's disease. Neuroscience 278, 354–366. 10.1016/j.neuroscience.2014.07.079 25168729

[B63] Ferrer I. (2009). Altered mitochondria, energy metabolism, voltage-dependent anion channel, and lipid rafts converge to exhaust neurons in Alzheimer's disease. J. Bioenergy Biomembr. 41, 425–431. 10.1007/s10863-009-9243-5 19798558

[B64] Fex Svenningsen A. Kanje M. (1999). Estrogen and progesterone stimulate Schwann cell proliferation in a sex- and age-dependent manner. J. Neurosci. Res. 57, 124–130. 10.1002/(SICI)1097-4547(19990701)57:1<124::AID-JNR13>3.0.CO;2-P 10397642

[B65] Galea L. A. Spritzer M. D. Barker J. M. Pawluski J. L. (2006). Gonadal hormone modulation of hippocampal neurogenesis in the adult. Hippocampus 16, 225–232. 10.1002/hipo.20154 16411182

[B66] Garcia-Estrada J. Del Rio J. A. Luquin S. Soriano E. Garcia-Segura L. M. (1993). Gonadal hormones down-regulate reactive gliosis and astrocyte proliferation after a penetrating brain injury. Brain Res. 628, 271–278. 10.1016/0006-8993(93)90964-O 8313156

[B67] Garcia-Segura L. M. Chowen J. A. Naftolin F. (1996). Endocrine glia: roles of glial cells in the brain actions of steroid and thyroid hormones and in the regulation of hormone secretion. Front. Neuroendocrinol. 17, 180–211. 10.1006/frne.1996.0005 8812295

[B68] Garcia-Segura L. M. Diz-Chaves Y. Perez-Martin M. Darnaudéry M. (2007). Estradiol, insulin-like growth factor-I and brain aging. Psychoneuroendocrinology 32, S57–S61. 10.1016/j.psyneuen.2007.03.001 17618061

[B69] Garcia-Segura L. M. Sanz A. Mendez P. (2006). Cross-talk between IGF-I and estradiol in the brain: focus on neuroprotection. Neuroendocrinology 84, 275–279. 10.1159/000097485 17124377

[B70] Garofalo T. Manganelli V. Grasso M. Mattei V. Ferri A. Misasi R.. (2015). Role of mitochondrial raft-like microdomains in the regulation of cell apoptosis. Apoptosis 20, 621–634. 10.1007/s10495-015-1100-x 25652700

[B71] George K. S. Wu S. (2012). Lipid raft: a floating island of death or survival. Toxicol. Appl. Pharmacol. 259, 311–319. 10.1016/j.taap.2012.01.007 22289360 PMC3299927

[B72] Giatti S. D'Intino G. Maschi O. Pesaresi M. Garcia-Segura L. M. Calza L.. (2010). Acute experimental autoimmune encephalomyelitis induces sex dimorphic changes in neuroactive steroid levels. Neurochem. Int. 56, 118–127. 10.1016/j.neuint.2009.09.009 19772882

[B73] Giatti S. Romano S. Pesaresi M. Cermenati G. Mitro N. Caruso D.. (2015). Neuroactive steroids and the peripheral nervous system: an update. Steroids 103, 23–30. 10.1016/j.steroids.2015.03.014 25824325 PMC6314841

[B74] Gilardi K. V. Shideler S. E. Valverde C. R. Roberts J. A. Lasley B. L. (1997). Characterization of the onset of menopause in the rhesus macaque. Biol. Reprod. 57, 335–340. 10.1095/biolreprod57.2.335 9241047

[B75] Giltay E. J. Gooren L. J. Toorians A. W. Katan M. B. Zock P. L. (2004). Docosahexaenoic acid concentrations are higher in women than in men because of estrogenic effects. Am. J. Clin. Nutr. 80, 1167–1174. 10.1093/ajcn/80.5.1167 15531662

[B76] Goedert M. (2015). Alzheimer's and Parkinson's diseases: the prion concept in relation to assembled Aβ, tau, and α-synuclein. Science 349:1255555. 10.1126/science.1255555 26250687

[B77] Greendale G. A. Ishii S. Huang M. H. Karlamangia A. S. (2013). Predicting the timeline to the final menstrual period: the study of women's health across the nation. J Clin. Endocrinol. Metab. 98, 1483–1491. 10.1210/jc.2012-3732 23533245 PMC3615211

[B78] Grodstein F. Chen J. Pollen D. A. Albert M. S. Wilson R. S. Folstein M. F.. (2000). Postmenopausal hormone therapy and cognitive function in healthy older. Am. Geriatr. Soc. 48, 746–752. 10.1111/j.1532-5415.2000.tb04748.x 10894312

[B79] Gupta R. (2017). Phosphorylation of rat brain purified mitochondrial voltage-dependent anion channel by c-Jun N-terminal kinase-3 modifies open-channel noise. Biochem. Biophys. Res. Commun. 490, 1221–1225. 10.1016/j.bbrc.2017.06.194 28676395

[B80] Han X. (2007). Potential mechanisms contributing to sulfatide depletion at the earliest clinically recognizable stage of Alzheimer's disease: a tale of shotgun lipidomics. J. Neurochem. 103, 171–179. 10.1111/j.1471-4159.2007.04708.x 17986152 PMC2147059

[B81] Hara Y. Waters E. M. McEwen B. S. Morrison J. H. (2015). Estrogen effects on cognitive and synaptic health over the lifecourse. Physiol. Rev. 95, 785–807. 10.1152/physrev.00036.2014 26109339 PMC4491541

[B82] Hara Y. Yuk F. Puri R. Janssen W. G. Rapp P. R. Morrison J. H. (2014). Presynaptic mitochondrial morphology in monkey prefrontal cortex correlates with working memory and is improved with estrogen treatment. Proc. Natl. Acad. Sci. U.S.A. 111, 486–491. 10.1073/pnas.1311310110 24297907 PMC3890848

[B83] Harlow S. D. Gass M. Hall J. E. Lobo R. Maki P. Rebar R. W.. (2012). Executive summary of the Stages of Reproductive Aging Workshop + 10: addressing the unfinished agenda of staging reproductive aging. Menopause 19, 387–395. 10.1097/gme.0b013e31824d8f40 22343510 PMC3340903

[B84] Hein L. K. Rozaklis T. Adams M. K. Hopwood J. J. Karageorgos L. (2017). Lipid composition of microdomains is altered in neuronopathic Gaucher disease sheep brain and spleen. Mol. Genet. Metab. 121, 259–270. 10.1016/j.ymgme.2017.05.010 28532689

[B85] Heldring N. Pike A. Andersson S. Matthews J. Cheng G. Hartman J.. (2007). Estrogen receptors: how do they signal and what are their targets. Physiol. Rev. 87, 905–931. 10.1152/physrev.00026.2006 17615392

[B86] Henderson V. W. (2014). Alzheimer's disease: review of hormone therapy trials and implications for treatment and prevention after menopause. J. Steroid Biochem. Mol. Biol. 142, 99–106. 10.1016/j.jsbmb.2013.05.010 23727128 PMC3830600

[B87] Herrera J. L. Diaz M. Hernández-Fernaud J. R. Salido E. Alonso R. Fernández C.. (2011a). Voltage-dependent anion channel as a resident protein of lipid rafts: post-transductional regulation by estrogens and involvement in neuronal preservation against Alzheimer's disease. J. Neurochem. 116, 820–827. 10.1111/j.1471-4159.2010.06987.x 21214547

[B88] Herrera J. L. Fernandez C. Diaz M. Cury D. Marin R. (2011b). Estradiol and tamoxifen differentially regulate a plasmalemmal voltage-dependent anion channel involved in amyloid-β induced neurotoxicity. Steroids 76, 840–844. 10.1016/j.steroids.2011.02.014 21354436

[B89] Hogervorst E. Williams J. Budge M. Riedel W. Jolles J. (2000). The nature of the effect of female gonadal hormone replacement therapy on cognitive function in post-menopausal women: a meta-analysis. Neuroscience 101, 485–512. 10.1016/S0306-4522(00)00410-3 11113299

[B90] Hojo Y. Murakami G. Mukai H. Higo S. Hatanaka Y. Ogiue-Ikeda M.. (2008). Estrogen synthesis in the brain–role in synaptic plasticity and memory. Mol. Cell. Endocrinol. 290, 31–43. 10.1016/j.mce.2008.04.017 18541362

[B91] Horia E. Watkins B. A. (2007). Complementary actions of docosahexaenoic acid and genistein on COX-2, PGE2 and invasiveness in MDA-MB-231 breast cancer cells. Carcinogenesis 28, 809–815. 10.1093/carcin/bgl183 17052999

[B92] Ishihara Y. Itoh K. Tanaka M. Tsuji M. Kawamoto T. Kawato S.. (2017). Potentiation of 17β-estradiol synthesis in the brain and elongation of seizure latency through dietary supplementation with docosahexaenoic acid. Sci. Rep. 7:6268. 10.1038/s41598-017-06630-0 28740157 PMC5524681

[B93] Ishunina T. A. Swaab D. F. (2008). Estrogen receptor-alpha splice variants in the human brain. Gynecol. Endocrinol. 24, 93–98. 10.1080/09513590701705148 18210333

[B94] Itoh N. Kim R. Peng M. DiFilippo E. Johnsonbaugh H. MacKenzie-Graham A.. (2017). Bedside to bench to bedside research: estrogen receptor beta ligand as a candidate neuroprotective treatment for multiple sclerosis. J. Neuroimmunol. 304, 63–71. 10.1016/j.jneuroim.2016.09.017 27771018 PMC5806698

[B95] Jin Y. Park Y. (2015). N-3 polyunsaturated fatty acids and 17β-estradiol injection induce antidepressant-like effects through regulation of serotonergic neurotransmission in ovariectomized rats. J. Nutr. Biochem. 26, 970–977. 10.1016/j.jnutbio.2015.04.005 26022074

[B96] Jung-Testas I. Renoir M. Bugnard H. Greene G. L. Baulieu E. E. (1992). Demonstration of steroid hormone receptors and steroid action in primary cultures of rat glial cells. J. Steroid Biochem. Mol. Biol. 41, 621–631. 10.1016/0960-0760(92)90394-X 1562533

[B97] Jung-Testas I. Schumacher M. Bugnard H. Baulieu E. E. (1993). Stimulation of rat Schwann cell proliferation by estradiol: synergism between the estrogen and cAMP. Brain Res. Dev. Brain Res. 72, 282–290. 10.1016/0165-3806(93)90194-F 8387406

[B98] Kazlauskaite J. Sanghera N. Sylvester I. Vénien-Bryan C. Pinheiro T. J. (2003). Structural changes of the prion protein in lipid membranes leading to aggregation and fibrillization. Biochemistry. 42, 3295–3304. 10.1021/bi026872q 12641461

[B99] Kelly M. J. Levin E. R. (2001). Rapid actions of plasma membrane estrogen receptors. Trends Endocrinol. Metab. 12, 152–156. 10.1016/S1043-2760(01)00377-0 11295570

[B100] Kim C. K. Torcaso A. Asimes A. Chung W. C. J. Pak T. R. (2017). Structural and functional characteristics of estrogen receptor beta., (ERβ) splice variants: implications for the aging brain. J. Neuroendocrinol. 30:e12488 10.1111/jne.12488 PMC5693782 28514502

[B101] Kimura D. (1995). Estrogen replacement therapy may protect against intellectual decline in postmenopausal women. Horm. Behav. 29, 312–321. 10.1006/hbeh.1995.1022 7490007

[B102] Kruger M. C. Tousen Y. Katsumata S. Tadaishi M. Kasonga A. E. Deepak V.. (2015). Effects of soy phytoestrogens and New Zealand functional foods on bone health. J Nutr. Sci. Vitaminol. 61(Suppl.), S142–S144. 10.3177/jnsv.61.S142 26598831

[B103] Kunzler J. Youmans K. L. Yu C. Ladu M. J. Tai L. M. (2014). APOE modulates the effect of estrogen therapy on Aβ accumulation EFAD-Tg mice. Neurosci. Lett. 560, 131–136. 10.1016/j.neulet.2013.12.032 24368217 PMC3955876

[B104] Lan Y. L. Zhao J. Li S. (2015). Update on the neuroprotective effect of estrogen receptor α against Alzheimer's disease. J Alzheimers Dis. 43, 1137–1148. 10.3233/JAD-141875 25159676

[B105] Lebesgue D. Chevaleyre V. Zukin R. S. Etgen A. M. (2009). Estradiol rescues neurons from global ischemia-induced cell death: multiple cellular pathways of neuroprotection. Steroids 74, 555–561. 10.1016/j.steroids.2009.01.003 19428444 PMC3029071

[B106] Ledesma M. D. Martin M. G. Dotti C. G. (2012). Lipid changes in the aged brain: effect on synaptic function and neuronal survival. Prog. Lipid Res. 51, 23–35. 10.1016/j.plipres.2011.11.004 22142854

[B107] Levental I. Veatch S. (2016). The continuing mystery of lipid rafts. J. Mol. Biol. 428, 4749–4764. 10.1016/j.jmb.2016.08.022 27575334 PMC5124408

[B108] Levental I. Grzybek M. Simons K. (2010). Greasing their way: lipid modifications determine protein association with membrane rafts. Biochemistry 49, 6305–6316. 10.1021/bi100882y 20583817

[B109] Levin E. R. (2009). Plasma membrane estrogen receptors. Trends Endocrinol. Metab. 20, 477–482. 10.1016/j.tem.2009.06.009 19783454 PMC3589572

[B110] Lingwood D. Simons K. (2010). Lipid rafts as a membrane-organizing principle. Science 327, 46–50. 10.1126/science.1174621 20044567

[B111] Li R. Shen Y. Yang L. B. Lue L. F. Finch C. Rogers J. (2000). Estrogen enhances uptake of amyloid beta-protein by microglia derived from the human cortex. J. Neurochem. 75, 1447-1454. 10.1046/j.1471-4159.2000.0751447.x 10987824

[B112] Liu P. Rudick M. Anderson R. G. (2002). Multiple functions of caveolin-1. J. Biol. Chem. 277, 41295–41298. 10.1074/jbc.R200020200 12189159

[B113] Losecaat Vermeer A. B. Riečansk,ý I. Eisenegger C. (2016). Competition, testosterone, and adult neurobehavioral plasticity. Prog. Brain Res. 229, 213–238. 10.1016/bs.pbr.2016.05.004 27926439

[B114] Lu C. L. Herndon C. (2017). New roles for neuronal estrogen receptors. Neurogastroenterol. Motil. 29, 1–7. 10.1111/nmo.13121 28597596

[B115] Luoma J. I. Boulware M. I. Mermelstein P. G. (2008). Caveolin proteins and estrogen signaling in the brain. Mol. Cell. Endocrinol. 290, 8–13. 10.1016/j.mce.2008.04.005 18502030 PMC2565274

[B116] Luquin S. Naftolin F. Garcia-Segura L. M. (1993). Natural fluctuation and gonadal hormone regulation of astrocyte immunoreactivity in dentate gyrus. J. Neurobiol. 24, 913–924. 10.1002/neu.480240705 8228969

[B117] Manson J. E. Chlebowski R. T. Stefanick M. L. Aragaki A. K. Rossouw J. E. Prentice R. L.. (2013). Menopausal hormone therapy and health outcomes during the intervention and extended poststopping phases of the Women's Health Initiative randomized trials. JAMA 310, 1353–1368. 10.1001/jama.2013.278040 24084921 PMC3963523

[B118] Marin R. (2011). Signalosomes in the brain: relevance in the development of certain neuropathologies such as Alzheimer's disease. Front. Physiol. 2:23. 10.3389/fphys.2011.00023 21852974 PMC3151622

[B119] Marin R. Casañas V. Pérez J. A. Fabelo N. Fernandez C. E. Diaz M. (2013b). Oestrogens as modulators of neuronal signalosomes and brain lipid homeostasis related to protection against neurodegeneration. J. Neuroendocrinol. 25, 1104–1115. 10.1111/jne.12068 23795744

[B120] Marin R. Díaz M. Alonso R. Sanz A. Arévalo M. A. Garcia-Segura L. M. (2009). Role of estrogen receptor α in membrane-initiated signaling in neural cells: interaction with IGF-1 receptor. J. Steroid Biochem. Mol. Biol. 114, 2–7. 10.1016/j.jsbmb.2008.12.014 19167493

[B121] Marin R. Fabelo N. Martín V. Garcia-Esparcia P. Ferrer I. Quinto-Alemany D.. (2017). Anomalies occurring in lipid profiles and protein distribution in frontal cortex lipid rafts in dementia with Lewy bodies disclose neurochemical traits partially shared by Alzheimer's and Parkinson's diseases. Neurobiol. Aging 49, 52–59. 10.1016/j.neurobiolaging.2016.08.027 27768960

[B122] Marin R. Guerra B. Alonso R. Ramírez C. M. Díaz M. (2005). Estrogen activates classical and alternative mechanisms to orchestrate neuroprotection. Curr. Neurovasc. Res. 2, 287–301. 10.2174/156720205774322629 16181121

[B123] Marin R. Marrero-Alonso J. Fernandez C. Cury D. Diaz M. (2012). Estrogen receptors in lipid raft signaling complexes for neuroprotection. Front. Biosci. 4, 1420–1433. 10.2741/e471 22201966

[B124] Marin R. Ramírez C. M. González M. Alonso R. Díaz M. (2006). Alternative estrogen receptors homologous to classical receptor alpha in murine neural tissues. Neurosci. Lett. 395, 7–11. 10.1016/j.neulet.2005.10.047 16288833

[B125] Marin R. Ramírez C. M. González M. González-Muñoz E. Zorzano A. Camps M.. (2007). Voltage-dependent anion channel., (VDAC) participates in amyloid β-induced toxicity and interacts with plasma membrane estrogen receptor α in septal and hippocampal neurons. Mol. Membr. Biol. 24, 148–160. 10.1080/09687860601055559 17453421

[B126] Marin R. Ramírez C. Morales A. González M. Alonso R. Díaz M. (2008). Modulation of Aβ-induced neurotoxicity by estrogen receptor alpha and other associated proteins in lipid rafts. Steroids 73, 992–996. 10.1016/j.steroids.2007.12.007 18242653

[B127] Marin R. Rojo J. A. Fabelo N. Fernandez C. E. Diaz M. (2013a). Lipid raft disarrangement as a result of neuropathological progresses: a novel strategy for early diagnosis? Neuroscience 245, 26–39. 10.1016/j.neuroscience.2013.04.025 23618758

[B128] Marin-Husstege M. Muggironi M. Raban D. Skoff R. P. Casaccia-Bonnefil P. (2004). Oligodendrocyte progenitor proliferation and maturation is differentially regulated by male and female sex steroid hormones. Dev. Neurosci. 26, 245–254. 10.1159/000082141 15711064

[B129] Marjoribanks J. Farquhar C. Roberts H. Lethaby A. Lee J. (2017). Long-term hormone therapy for perimenopausal and postmenopausal women. Cochrane Database Syst. Rev. 1:CD004143. 10.1002/14651858.CD004143.pub5 28093732 PMC6465148

[B130] Martín V. Fabelo N. Santpere G. Puig B. Marín R. Ferrer I.. (2010). Lipid alterations in lipid rafts from Alzheimer's disease human brain cortex. J. Alzheimers. Dis. 19, 489–502. 10.3233/JAD-2010-1242 20110596

[B131] Maselli A. Pierdominici M. Vitale C. Ortona E. (2015). Membrane lipid rafts and estrogenic signaling: a functional role in the modulation of cell homeostasis. Apoptosis 20, 671–678. 10.1007/s10495-015-1093-5 25637184

[B132] Massimino M. L. Griffoni C. Spisni E. Toni M. Tomasi V. (2002). Involvement of caveolae and caveolae-like domains in signalling, cell survival and angiogenesis. Cell Signal. 14, 93–98. 10.1016/S0898-6568(01)00232-7 11781132

[B133] McCarthy M. M. (2008). Estradiol and the developing brain. Physiol. Rev. 88, 91–124. 10.1152/physrev.00010.2007 18195084 PMC2754262

[B134] McEwen B. S. Akama K. T. Spencer-Segal J. L. Milner T. A. Waters E. M. (2012). Estrogen effects on the brain: actions beyond the hypothalamus via novel mechanisms. Behav. Neurosci. 126, 4–16. 10.1037/a0026708 22289042 PMC3480182

[B135] McGregor C. Riordan A. Thornton J. (2017). Estrogens and the cognitive symptoms of schizophrenia: possible neuroprotective mechanisms. Front. Neuroendocrinol. 47, 19–33. 10.1016/j.yfrne.2017.06.003 28673758

[B136] McNamara R. K. Able J. Jandacek R. Rider T. Tso P. (2009). Gender differences in rat erythrocyte and brain docosahexaenoic acid composition: role of ovarian hormones and dietary omega-3 fatty acid composition. Psychoneuroendocrinology 34, 532–539. 10.1016/j.psyneuen.2008.10.013 19046819 PMC2692269

[B137] Meitzen J. Mermelstein P. G. (2011). Estrogen receptors stimulate brain region specific metabotropic glutamate receptors to rapidly initiate signal transduction pathways. J. Chem. Neuroanat. 42, 236–241. 10.1016/j.jchemneu.2011.02.002 21458561 PMC3146967

[B138] Meitzen J. Luoma J. I. Boulware M. I. Hedges V. L. Peterson B. M. Tuomela K.. (2013). Palmitoylation of estrogen receptors is essential for neuronal membrane signaling. Endocrinology 154, 4293–4304. 10.1210/en.2013-1172 24008343 PMC3800757

[B139] Melcangi R. C. Garcia-Segura L. M. Mensah-Nyagan A. G. (2008). Neuroactive steroids: state of the art and new perspectives. Cell. Mol. Life Sci. 65, 777–797. 10.1007/s00018-007-7403-5 18038216 PMC11131680

[B140] Melcangi R. C. Giatti S. Garcia-Segura L. M. (2016). Levels and actions of neuroactive steroids in the nervous system under physiological and pathological conditions: sex-specific features. Neurosci. Biobehav. Rev. 67, 25–40. 10.1016/j.neubiorev.2015.09.023 26657814

[B141] Merlo S. Spampinato S. F. Sortino M. A. (2017). Estrogen and Alzheimer's disease: still an attractive topic despite disappointment from early clinical results. Eur. J. Pharmacol. 817, 51–58. 10.1016/j.ejphar.2017.05.059 28577965

[B142] Micevych P. E. Kelly M. J. (2012). Membrane estrogen receptor regulation of hypothalamic function. Neuroendocrinology 96, 103–110. 10.1159/000338400 22538318 PMC3496782

[B143] Micevych P. E. Mermelstein P. G. (2008). Membrane estrogen receptors acting through metabotropic glutamate receptors: an emerging mechanism of estrogen action in brain. Mol. Neurobiol. 38, 66–77. 10.1007/s12035-008-8034-z 18670908 PMC2663000

[B144] Miller K. J. Conney J. C. Rasgon N. L. Fairbanks L. A. Small G. W. (2002). Mood symptoms and cognitive performance in women estrogen users and nonusers and men. J. Am. Geriatr. 50, 1826–1830. 10.1046/j.1532-5415.2002.50511.x 12410901

[B145] Milner T. A. McEwen B. S. Hayashi S. Li C. J. Reagan L. P. Alves S. E. (2001). Ultrastructural evidence that hippocampal alpha estrogen receptors are located at extranuclear sites. J. Comp Neurol. 429, 355–371. 10.1002/1096-9861(20010115)429:3<355::AID-CNE1>3.0.CO;2-# 11116225

[B146] Moffat S. D. Zonderman A. B. Metter E. J. Kawas C. Blackman M. R. Harman S. M.. (2004). Free testosterone and risk for Alzheimer disease in older men. Neurology 62, 188–193. 10.1212/WNL.62.2.188 14745052

[B147] Molander-Melin M. Blennow K. Bogdanovic N. Dellheden B. Månsson J. E. Fredman P. (2005). Structural membrane alterations in Alzheimer brains found to be associated with regional disease development; increased density of gangliosides GM1 and GM2 and loss of cholesterol in detergent-resistant membrane domains. J. Neurochem. 92, 171–182. 10.1111/j.1471-4159.2004.02849.x 15606906

[B148] Murakami G. Hojo Y. Kato A. Komatsuzaki Y. Horie S. Soma M. (2017). Rapid non-genomic modulation by neurosteroids of dendritic spines in the hippocampus: androgen, estrogen and corticosteroid. J. Neuroendocrinol. 30:e12561 10.1111/jne.12561 29194818

[B149] Nathan B. P. Barsukova A. G. Shen F. McAsey M. Struble R. G. (2004). Estrogen facilitates neurite extension via apolipoprotein E in cultured adult mouse cortical neurons. Endocrinology 145, 3065–3073. 10.1210/en.2003-1707 15033916

[B150] Neu S. C. Pa J. Kukull W. Beekly D. Kuzma A. Gangadharan P.. (2017). Apolipoprotein E genotype and sex risk factors for Alzheimer Disease: a meta-analysis. JAMA Neurol. 74, 1178–1189. 10.1001/jamaneurol.2017.2188 28846757 PMC5759346

[B151] Nilsen J. Irwin R. W. Gallaher T. K. Brinton R. D. (2007). Estradiol *in vivo* regulation of brain mitochondrial proteome. J. Neurosci. 27, 14069–14077. 10.1523/JNEUROSCI.4391-07.2007 18094246 PMC6673510

[B152] Ogiue-Ikeda M. Tanabe N. Mukai H. Hojo Y. Murakami G. Tsurugizawa T.. (2008). Rapid modulation of synaptic plasticity by estrogens as well as endocrine disrupters in hippocampal neurons. Brain Res. Rev. 57, 363–375. 10.1016/j.brainresrev.2007.06.010 17822775

[B153] Osterlund M. K. Grandien K. Keller E. Hurd Y. L. (2000a). The human brain has distinct regional expression patterns of estrogen receptor alpha mRNA isoforms derived from alternative promoters. J. Neurochem. 75, 1390–1397. 10.1046/j.1471-4159.2000.0751390.x 10987818

[B154] Osterlund M. K. Gustafsson J. A. Keller E. Hurd Y. L. (2000b). Estrogen receptor β (ERbeta) messenger ribonucleic acid., (mRNA) expression within the human forebrain: distinct distribution pattern to ERalpha mRNA. J. Clin. Endocrinol. Metab. 85, 3840–3846. 10.1210/jcem.85.10.6913 11061547

[B155] Overk C. R. Perez S. E. Ma C. Taves M. D. Soma K. K. Mufson E. J. (2013). Sex steroid levels and AD-like pathology in 3xTgAD mice. J. Neuroendocrinol. 25, 131–144. 10.1111/j.1365-2826.2012.02374.x 22889357 PMC4065422

[B156] Panzica G. Melcangi R. C. (2016). Structural and molecular brain sexual differences: a tool to understand sex differences in health and disease. Neurosci. Biobehav. Rev. 67, 2–8. 10.1016/j.neubiorev.2016.04.017 27113294

[B157] Paratcha G. Ibáñez C. F. (2002). Lipid rafts and the control of neurotrophic factor signaling in the nervous system: variations on a theme. Curr. Opin. Neurobiol. 12, 542–549. 10.1016/S0959-4388(02)00363-X 12367633

[B158] Parsons R. B. Austen B. M. (2007). Protein-protein interactions in the assembly and subcellular trafficking of the BACE., (beta-site amyloid precursor protein-cleaving enzyme) complex of Alzheimer's disease. Biochem. Soc. Trans. 35, 974–979. 10.1042/BST0350974 17956258

[B159] Pawlak J. Karolczak M. Krust A. Chambon P. Beyer C. (2005). Estrogen receptor-alpha is associated with the plasma membrane of astrocytes and coupled to the MAP/Src-kinase pathway. Glia 50, 270–275. 10.1002/glia.20162 15712205

[B160] Pawlosky R. J. Hibbeln J. R. Novotny J. A. Salem N. Jr. (2001). Physiological compartmental analysis of alpha-linolenic acid metabolism in adult humans. J. Lipid Res. 42, 1257–1265. Available online at: http://www.jlr.org/content/42/8/1257.long 11483627

[B161] Pedram A. Razandi M. Sainson R. C. Kim J. K. Hughes C. C. Levin E. R. (2007). A conserved mechanism for steroid receptor translocation to the plasma membrane. J. Biol. Chem. 282, 22278–22288. 10.1074/jbc.M611877200 17535799

[B162] Pellegrini M. Pallottini V. Marin R. Marino M. (2014). Role of the sex hormone estrogen in the prevention of lipid disorder. Curr. Med. Chem. 21, 2734–2742. 10.2174/0929867321666140303123602 24606523

[B163] Peri A. (2016). Neuroprotective effects of estrogens: the role of cholesterol. J. Endocrinol. Invest. 39, 11–18. 10.1007/s40618-015-0332-5 26084445

[B164] Peri A. Benvenuti S. Luciani P. Deledda C. Cellai I. (2011). Membrane cholesterol as a mediator of the neuroprotective effects of estrogens. Neuroscience 191, 107–117. 10.1016/j.neuroscience.2011.03.011 21396986

[B165] Petrone A. B. Simpkins J. W. Barr T. L. (2014). 17β-estradiol and inflammation: implications for ischemic stroke. Aging Dis. 5, 340–345. 10.14336/AD.2014.0500340 25276492 PMC4173799

[B166] Petrovska S. Dejanova B. Jurisic V. (2012). Estrogens: mechanisms of neuroprotective effects. J. Physiol. Biochem. 68, 455–460. 10.1007/s13105-012-0159-x 22371015

[B167] Picillo M. Nicoletti A. Fetoni V. Garavaglia B. Barone P. Pellecchia M. T. (2017). The relevance of gender in Parkinson's disease: a review. J. Neurol. 264, 1583–1607. 10.1007/s00415-016-8384-9 28054129

[B168] Pietras R. J. Szego C. M. (1977). Specific binding sites for oestrogen at the outer surfaces of isolated endometrial cells. Nature 265, 69–72. 10.1038/265069a0 834244

[B169] Pike C. F. (2017). Sex and the development of Alzheimer's disease. J. Neurosci. Res. 95, 671–680. 10.1002/jnr.23827 27870425 PMC5120614

[B170] Platania P. Laureanti F. Bellomo M. Giuffrida R. Giuffrida-Stella A. M. Catania M. V.. (2003). Differential expression of estrogen receptors α and β in the spinal cord during postnatal development: localization in glial cells. Neuroendocrinology 77, 334–340. 10.1159/000070899 12806179

[B171] Plourde M. Cunnane S. C. (2007). Extremely limited synthesis of long chain polyunsaturates in adults: implications for their dietary essentiality and use as supplements. Appl. Physiol. Nutr. Metab. 32, 619–634. 10.1139/H07-034 17622276

[B172] Prentice R. L. (2014). Postmenopausal hormone therapy and the risks of coronary heart disease, breast cancer, and stroke. Semin. Reprod. Med. 32, 419–425. 10.1055/s-0034-1384624 25321418 PMC4212810

[B173] Pristerá A. Okuse K. (2012). Building excitable membranes: lipid rafts and multiple controls on trafficking of electrogenic molecules. Neuroscientist 18, 70–81. 10.1177/1073858410393977 21518816

[B174] Prokai L. Prokai-Tatrai K. Perjesi P. Zharikova A. D. Perez E. J. Liu R.. (2003). Quinol-based cyclic antioxidant mechanism in estrogen neuroprotection. Proc. Natl. Acad. Sci. U.S.A. 100, 11741–11746. 10.1073/pnas.2032621100 14504383 PMC208828

[B175] Prossintz E. R. Barton M. (2011). The G-protein-coupled estrogen receptor GPER in health and disease. Nat. Rev. Endocrinol. 7, 715–726. 10.1038/nrendo.2011.122 21844907 PMC3474542

[B176] Puskas L. G. Kitajka K. (2006). Nutrigenomic approaches to study the effects of n-3 PUFA diet in the central nervous system. Nutr. Health 18, 227–232. 10.1177/026010600601800305 17180868

[B177] Quesada A. Romeo H. E. Micevych P. (2007). Distribution and localization patterns of estrogen receptor-beta and insulin-like growth factor-1 receptors in neurons and glial cells of the female rat substantia nigra: localization of ERbeta and IGF-1R in substantia nigra. J. Comp. Neurol. 503, 198–208. 10.1002/cne.21358 17480015 PMC2907103

[B178] Raghava N. Das B. C. Ray S. K. (2017). Neuroprotective effects of estrogen in CNS injuries: insights from animal models. Neurosci. Neuroecon. 6, 15–29. 10.2147/NAN.S105134 28845391 PMC5567743

[B179] Ramagopalan S. V. Dobson R. Meier U. C. Giovannoni G. (2010). Multiple sclerosis: risk factors, prodromes, and potential causal pathways. Lancet Neurol. 9, 727–739. 10.1016/S1474-4422(10)70094-6 20610348

[B180] Ramirez C. M. González M. Díaz M. Alonso R. Ferrer I. Santpere G.. (2009). VDAC and ERalpha interaction in caveolae from human cortex is altered in Alzheimer's disease. Mol. Cell. Neurosci. 42, 172–183. 10.1016/j.mcn.2009.07.001 19595769

[B181] Rapoport S. (2013). Translational studies on regulation of brain docosahexaenoic acid., (DHA) *in vivo*. Prostaglandins Leukot. Essent. Fatty Acids 88, 79–85. 10.1016/j.plefa.2012.05.003 22766388 PMC3467358

[B182] Rasgon N. L. Silverman D. Siddarth P. Miller K. Ercoli L. M. Elman S.. (2005). Estrogen use and brain metabolic change in postmenopausal women. Neurobiol. Aging 26, 229–235. 10.1016/j.neurobiolaging.2004.03.003 15582750

[B183] Rawicz W. Olbrich K. C. McIntosh T. Needham D. Evans E. (2000). Effect of chain length and unsaturation on elasticity of lipid bilayers. Biophys. J. 79, 328–339. 10.1016/S0006-3495(00)76295-3 10866959 PMC1300937

[B184] Ray S. Kassan A. Busija A. R. Rangamani P. Patel H. H. (2016). The plasma membrane as a capacitor for energy and metabolism. Am. J. Physiol. Cell Physiol. 310, C181–C192. 10.1152/ajpcell.00087.2015 26771520 PMC4888523

[B185] Reeves M. J. Bushnell C. D. Howard G. Gargano J. W. Duncan P. W. Lynch G.. (2008). Sex differences in stroke: epidemiology, clinical presentation, medical care, and outcomes. Lancet Neurol. 7, 915–926. 10.1016/S1474-4422(08)70193-5 18722812 PMC2665267

[B186] Robinson D. Friedman L. Marcus R. Tinklenberg J. Yesavage J. (1994). Estrogen replacement therapy and memory in older women. J. Am. Geriatr. Soc. 42, 919–922. 10.1111/j.1532-5415.1994.tb06580.x 8064097

[B187] Rocca W. A. Grossardt B. R. Maraganore D. M. (2008). The long-term effects of oophorectomy on cognitive and motor aging are age dependent. Neurodegener. Dis. 5, 257–260. 10.1159/000113718 18322406 PMC2768565

[B188] Rosario E. R. Chang L. Head E. H. Stanczyk F. Z. Pike C. J. (2011). Brain levels of sex steroid hormones in men and women during normal aging and in Alzheimer's disease. Neurobiol. Aging 32, 604–613. 10.1016/j.neurobiolaging.2009.04.008 19428144 PMC2930132

[B189] Ryan J. Scali J. Carrière I. Amieva H. Rouaud O. Berr C.. (2014). Impact of a premature menopause on cognitive function in later life. BJOG 121, 1729–1739. 10.1111/1471-0528.12828 24802975

[B190] Salem N. Jr. Vandal M. Calon F. (2015). The benefit of docosahexaenoic acid for the adult brain in aging and dementia. Prostaglandins Leukot. Essent. Fatty Acids 92, 15–22. 10.1016/j.plefa.2014.10.003 25457546

[B191] Samuel F. Flavin W. P. Iqbal S. Pacelli C. Sri Renganathan S. D. Trudeau L. E.. (2016). Effects of Serine 129 phosphorylation on α-synuclein aggregation, membrane association, and internalization. J. Biol. Chem. 291, 4374–4385. 10.1074/jbc.M115.705095 26719332 PMC4813466

[B192] Schumacher M. Weill-Engerer S. Liere P. Robert F. Franklin R. J. Garcia-Segura L. M.. (2003). Steroid hormones and neurosteroids in normal and pathological aging of the nervous system. Prog. Neurobiol. 71, 3–29. 10.1016/j.pneurobio.2003.09.004 14611864

[B193] Schreihofer D. A. Ma Y. (2013). Estrogen receptors and ischemic neuroprotection: who, what, where, and when? Brain Res. 1514, 107–122. 10.1016/j.brainres.2013.02.051 23500634

[B194] Sebastião A. M. Colino-Oliveira M. Assaife-Lopes N. Dias R. B. Ribeiro J. A. (2013). Lipid rafts, synaptic transmission and plasticity: impact in age-related neurodegenerative diseases. Neuropharmacology 64, 97–107. 10.1016/j.neuropharm.2012.06.053 22820274

[B195] Shaikh S. R. Wassall S. R. Brown D. A. Kosaraju R. (2015). N-3 polyunsaturated fatty acids, lipid microclusters, and vitamin, E. Curr. Top. Membr. 75, 209–231. 10.1016/bs.ctm.2015.03.003 26015284

[B196] Sheppard P. A. S. Koss W. A. Frick K. M. Choleris E. (2017). Rapid actions of estrogens and their receptors on memory acquisition and consolidation in females. J. Neuroendocrinol. 30:e12485 10.1111/jne.12485 PMC6543823 28489296

[B197] Sherwin B. B. Henry J. F. (2008). Brain aging modulates the neuroprotective effects of estrogen on selective aspects of cognition in women: a critical review. Front. Neuroendocrinol. 29, 88–113. 10.1016/j.yfrne.2007.08.002 17980408

[B198] Shi L. Du X. Zhou H. Tao C. Liu Y. Meng F.. (2014). Cumulative effects of the ApoE genotype and gender on the synaptic proteome and oxidative stress in the mouse brain. Int. J. Neuropsychopharmacol. 17, 1863–1879. 10.1017/S1461145714000601 24810422

[B199] Siddiqui A. N. Siddiqui N. Khan R. A. Kalam A. Jabir N. R. Kamal M. A.. (2016). Neuroprotective role of steroidal sex hormones: an overview. CNS Neurosci. Ther. 22, 342–350. 10.1111/cns.12538 27012165 PMC6492877

[B200] Sierra A. Gottfried-Blackmore A. Milner T. A. McEwen B. S. Bulloch K. (2008). Steroid hormone receptor expression and function in microglia. Glia 56, 659–674. 10.1002/glia.20644 18286612

[B201] Simons K. Gerl M. J. (2010). Revitalizing membrane rafts: new tools and insights. Nat. Rev. Mol. Cell Biol. 11, 688–699. 10.1038/nrm2977 20861879

[B202] Simpkins J. W. Dykens J. A. (2008). Mitochondrial mechanisms of estrogen neuroprotection. Brain Res. Rev. 57, 421–430. 10.1016/j.brainresrev.2007.04.007 17512984

[B203] Sinclair A. J. Begg D. Mathai M. Weisinger R. S. (2007). Omega 3 fatty acids and the brain: review of studies in depression. Asia Pac. J. Clin. Nutr. 16, 391–397. 17392137

[B204] Söderberg M. Edlund C. Kristensson K. Dallner G. (1991). Fatty acid composition of brain phospholipids in aging and in Alzheimer's disease. Lipids 26, 421–425. 10.1007/BF02536067 1881238

[B205] Soni M. Rahardjo T. B. Soekardi R. Sulistyowati Y. Lestariningsih Yesufu-Udechuku, A.. (2014). Phytoestrogens and cognitive function: a review. Maturitas 77, 209–220. 10.1016/j.maturitas.2013.12.010 24486046

[B206] Sonnino S. Aureli M. Grassi S. Mauri L. Prioni S. Prinetti A. (2014). Lipid rafts in neurodegeneration and neuroprotection. Mol. Neurobiol. 50, 130–148. 10.1007/s12035-013-8614-4 24362851

[B207] Srivastava D. P. Waters E. M. Mermelstein P. G. Kramár E. A. Shors T. J. Liu F. (2011). Rapid estrogen signaling in the brain: implications for the fine-tuning of neuronal circuitry. J. Neurosci. 31, 16056–16063. 10.1523/JNEUROSCI.4097-11.2011 22072656 PMC3245715

[B208] Stillwell W. Wassall S. R. (2003). Docosahexaenoic acid: membrane properties of a unique fatty acid. Chem. Phys. Lipids 126, 1–27. 10.1016/S0009-3084(03)00101-4 14580707

[B209] Su H. M. (2010). Mechanisms of n-3 fatty acid-mediated development and maintenance of learning memory performance. J. Nutr. Biochem. 21, 364–373. 10.1016/j.jnutbio.2009.11.003 20233652

[B210] Sun G. Y. Simonyi A. Fritsche K. L. Chuang D. Y. Hannink M. Gu Z.. (2017). Docosahexaenoic acid., (DHA): An essential nutrient and a nutraceutical for brain health and diseases. Prostaglandins Leukot. Essent. Fatty Acids. [Epub ahead of print]. 10.1016/j.plefa.2017.03.006 28314621 PMC9087135

[B211] Takahashi N. Tonchev A. B. Koike K. Murakami K. Yamada K. Yamashima T.. (2004). Expression of estrogen receptor-beta in the postischemic monkey hippocampus. Neurosci. Lett. 369, 9–13. 10.1016/j.neulet.2004.07.042 15380298

[B212] Tapia-Gonzalez S. Carrero P. Pernia O. Garcia-Segura L. M. Diz-Chaves Y. (2008). Selective oestrogen receptor., (ER) modulators reduce microglia reactivity *in vivo* after peripheral inflammation: potential role of microglial ERs. J. Endocrinol. 198, 219–230. 10.1677/JOE-07-0294 18460549

[B213] Taylor D. R. Hooper N. M. (2006). The prion protein and lipid rafts. Mol. Membr. Biol. 23, 89–99. 10.1080/09687860500449994 16611584

[B214] Thaung Zaw J. J. Howe P. R. C. Wong R. H. X. (2017). Does phytoestrogen supplementation improve cognition in humans? A systematic review. Ann. N. Y. Acad. Sci. 1403, 150–163. 10.1111/nyas.13459 28945939

[B215] Thinnes F. P. (2013). New findings concerning vertebrate porin II–on the relevance of glycine motifs of type-1 VDAC. Mol. Genet. Metab. 108, 212–224. 10.1016/j.ymgme.2013.01.008 23419876

[B216] Thinnes F. P. (2015). After all, plasmalemmal expression of type-1 VDAC can be understood. Phosphorylation, nitrosylation, and channel modulators work together in vertebrate cell volume regulation and either apoptotic pathway. Front. Physiol. 6:126. 10.3389/fphys.2015.00126 25964761 PMC4410597

[B217] Tiwari-Woodruff S. Voskuhl R. R. (2009). Neuroprotective and anti-inflammatory effects of estrogen receptor ligand treatment in mice. J. Neurol. Sci. 286, 81–85. 10.1016/j.jns.2009.04.023 19442988 PMC2760614

[B218] Tiwari-Woodruff S. Morales L. B. Lee R. Voskuhl R. R. (2007). Differential neuroprotective and antiinflammatory effects of estrogen receptor., (ER)α and ERβ ligand treatment. Proc. Natl. Acad. Sci. U.S.A. 104, 14813–14818. 10.1073/pnas.0703783104 17785421 PMC1976208

[B219] Toonen J. A. Solga A. C. Ma Y. Gutmann D. H. (2017). Estrogen activation of microglia underlies the sexually dimorphic differences in Nf1 optic glioma-induced retinal pathology. J. Exp. Med. 214, 17–25. 10.1084/jem.20160447 27923908 PMC5206494

[B220] Tsui-Pierchala B. A. Encinas M. Milbrandt J. Johsson E. M. Jr. (2002). Lipid rafts in neuronal signaling and function. Trends Neurosci. 25, 412–417. 10.1016/S0166-2236(02)02215-4 12127758

[B221] Ugalde C. L. Finkelstein D. I. Lawson V. A. Hill A. F. (2016). Pathogenic mechanisms of prion protein, amyloid-β and α-synuclein misfolding: the prion concept and neurotoxicity of protein oligomers. J. Neurochem. 139, 162–180. 10.1111/jnc.13772 27529376

[B222] Valencia A. Reeves P. B. Sapp E. Li X. Alexander J. Kegel K. B.. (2010). Mutant huntingtin and glycogen synthase kinase 3-beta accumulate in neuronal lipid rafts of a presymptomatic knock-in mouse model of Huntington's disease. J. Neurosci. Res. 88, 179–190. 10.1002/jnr.22184 19642201

[B223] Valverde M. A. Hardy S. P. Díaz M. (2002). Activation of Maxi Cl(-) channels by antiestrogens and phenothiazines in NIH3T3 fibroblasts. Steroids 67, 439–445. 10.1016/S0039-128X(01)00174-X 11960619

[B224] Van Kempen T. A. Gorecka J. Gonzalez A. D. Soeda F. Milner T. A. Waters E. M. (2014). Characterization of neural estrogen signaling and neurotrophic changes in the accelerated ovarian failure mouse model of menopause. Endocrinology 155, 3610–3623. 10.1210/en.2014-1190 24926825 PMC4138565

[B225] Vegeto E. Belcredito S. Ghisletti S. Meda C. Etteri S. Maggi A. (2006). The endogenous estrogen status regulates microglia reactivity in animal models of neuroinflammation. Endocrinology 147, 2263–2272. 10.1210/en.2005-1330 16469811

[B226] Vellas B. Carrie I. Gillette-Guyonnet S. Touchon J. Dantoine T. Dartigues J. F.. (2014). MAPT study: a multidomain approach for preventing Alzheimer's disease: design and baseline data. J. Prev. Alzheimers Dis. 1, 13–22. 26594639 PMC4652787

[B227] Vetrivel K. S. Meckler X. Chen Y. Nguyen P. D. Seidah N. G. Vassar R.. (2009). Alzheimer disease Abeta production in the absence of S-palmitoylation-dependent targeting of BACE1 to lipid rafts. J. Biol. Chem. 284, 3793–3803. 10.1074/jbc.M808920200 19074428 PMC2635050

[B228] Villa P. Amar I. D. Bottoni C. Cipolla C. Dinoi G. Moruzzi M. C.. (2017). The impact of combined nutraceutical supplementation on quality of life and metabolic changes during the menopausal transition: a pilot randomized trial. Arch. Gynecol. Obstet. 296, 791–801. 10.1007/s00404-017-4491-9 28852842

[B229] Walker M. L. Herndon J. G. (2008). Menopause in nonhuman primates? Biol. Reprod. 79, 398–406. 10.1095/biolreprod.108.068536 18495681 PMC2553520

[B230] Wang Z. F. Pan Z. Y. Xu C. S. Li Z. Q. (2017). Activation of G-protein coupled estrogen receptor 1 improves early-onset cognitive impairment via PI3K/Akt pathway in rats with traumatic brain injury. Biochem. Biophys. Res. Commun. 482, 948–953. 10.1016/j.bbrc.2016.11.138 27908726

[B231] Wassall S. R. Stillwell W. (2009). Polyunsaturated fatty acid-cholesterol interactions: domain formation in membranes. Biochim. Biophys. Acta 1788, 24–32. 10.1016/j.bbamem.2008.10.011 19014904

[B232] Wooten G. F. Currie L. J. Bovbjerg V. E. Lee J. K. Patrie J. (2004). Are men at greater risk for Parkinson's disease than women? J. Neurol. Neurosurg. Psychiatr. 75, 637–639. 10.1136/jnnp.2003.020982 15026515 PMC1739032

[B233] Yanagisawa K. (2002). Cholesterol and pathological processes in Alzheimer's disease. J. Neurosci. Res. 70, 361–366. 10.1002/jnr.10348 12391598

[B234] Yang S. H. Sarkar S. N. Liu R. Perez E. J. Wang X. Wen Y.. (2009). Estrogen receptor beta as a mitochondrial vulnerability factor. J. Biol. Chem. 284, 9540–9548. 10.1074/jbc.M808246200 19189968 PMC2666606

[B235] Yassine H. N. Feng Q. Azizkhanian I. Rawat V. Castor K. Fonteh A. N.. (2016). Association of serum docosahexaenoic acid with cerebral amyloidosis. JAMA Neurol. 73, 1208–1216. 10.1001/jamaneurol.2016.1924 27532692

[B236] Yue X. Lu M. Lancaster T. Cao P. Honda S. Staufenbiel M.. (2005). Brain estrogen deficiency accelerates Aβ plaque formation in an Alzheimer's disease animal model. Proc. Natl. Acad. Sci. U.S.A. 102, 19198–19203. 10.1073/pnas.0505203102 16365303 PMC1323154

[B237] Yurko-Mauro K. McCarthy D. Rom D. Nelson E. B. Ryan A. S. Blackwell A.. (2010). Beneficial effects of docosahexaenoic acid on cognition in age-related cognitive decline. Alzheimers. Dement.. 6, 456–464. 10.1016/j.jalz.2010.01.013 20434961

[B238] Zárate R. El Jaber-Vazdekis N. Tejera N. Pérez J. A. Rodríguez C. (2017). Significance of long chain polyunsaturated fatty acids in human health. Clin. Transl. Med. 6:25. 10.1186/s40169-017-0153-6 28752333 PMC5532176

[B239] Zhang L. Li B. S. Zhao W. Chang Y. H. Ma W. Dragan M.. (2002). Sex-related differences in MAPKs activation in rat astrocytes: effects of estrogen on cell death. Brain Res. Mol. Brain Res. 103, 1–11. 10.1016/S0169-328X(02)00130-4 12106687

[B240] Zhao L. O'Neill K. Diaz Brinton R. (2005). Selective estrogen receptor modulators., (SERMs) for the brain: current status and remaining challenges for developing NeuroSERMs. Brain Res. Brain Res. Rev. 49, 472–493. 10.1016/j.brainresrev.2005.01.009 16269315

[B241] Zhao T. Z. Shi F. Hu J. He S. M. Ding Q. Ma L. T. (2016). GPER1 mediates estrogen-induced neuroprotection against oxygen-glucose deprivation in the primary hippocampal neurons. Neuroscience 328, 117–126. 10.1016/j.neuroscience.2016.04.026 27113328

